# Supplementary material for: Intelectin-2 is a broad-spectrum antimicrobial lectin
Source: Nat Commun. 2026 Jan 13;17:231. doi: 10.1038/s41467-025-67099-4 (PMC12800186; doi:10.1038/s41467-025-67099-4)
Supplement: Supplementary file 1 — Supplementary Information [file 41467_2025_67099_MOESM1_ESM.pdf]

Supplementary Information

Intelectin-2 is a broad-spectrum antimicrobial lectin

Authors: Amanda E. Dugan[1,9], Deepsing Syangtan[1,9], Eric B. Nonnecke[2], Rajeev S. Chorghade[1], Amanda L. Peiffer[1], Jenny J. Yao[1], Jessie Ille-Bunn[1], Dallis Sergio[3], Gleb Pishchany[3], Catherine Dhennezel[3], Hera Vlamakis[3], Sunhee Bae[1], Sheila Johnson[4], Chariesse Ellis[4], Soumi Ghosh[5], Jill W. Alty[1], Carolyn E. Barnes[1], Miri Krupkin[6], Gerardo Cárcamo-Oyarce [6], Katharina Ribbeck[6], Ramnik J. Xavier[3,7], Charles L. Bevins[2], and Laura L. Kiessling[1,3,4,8,10,*]

The PDF file includes:

Supplementary Figures 1 to 12
Supplementary Table 1
Source Data for Supplementary Figures

Other Supplementary Materials for this manuscript include the following:

Supplementary Tables 2 and 3 (Separate files)
Supplementary Movies 1 to 3 (Separate files)

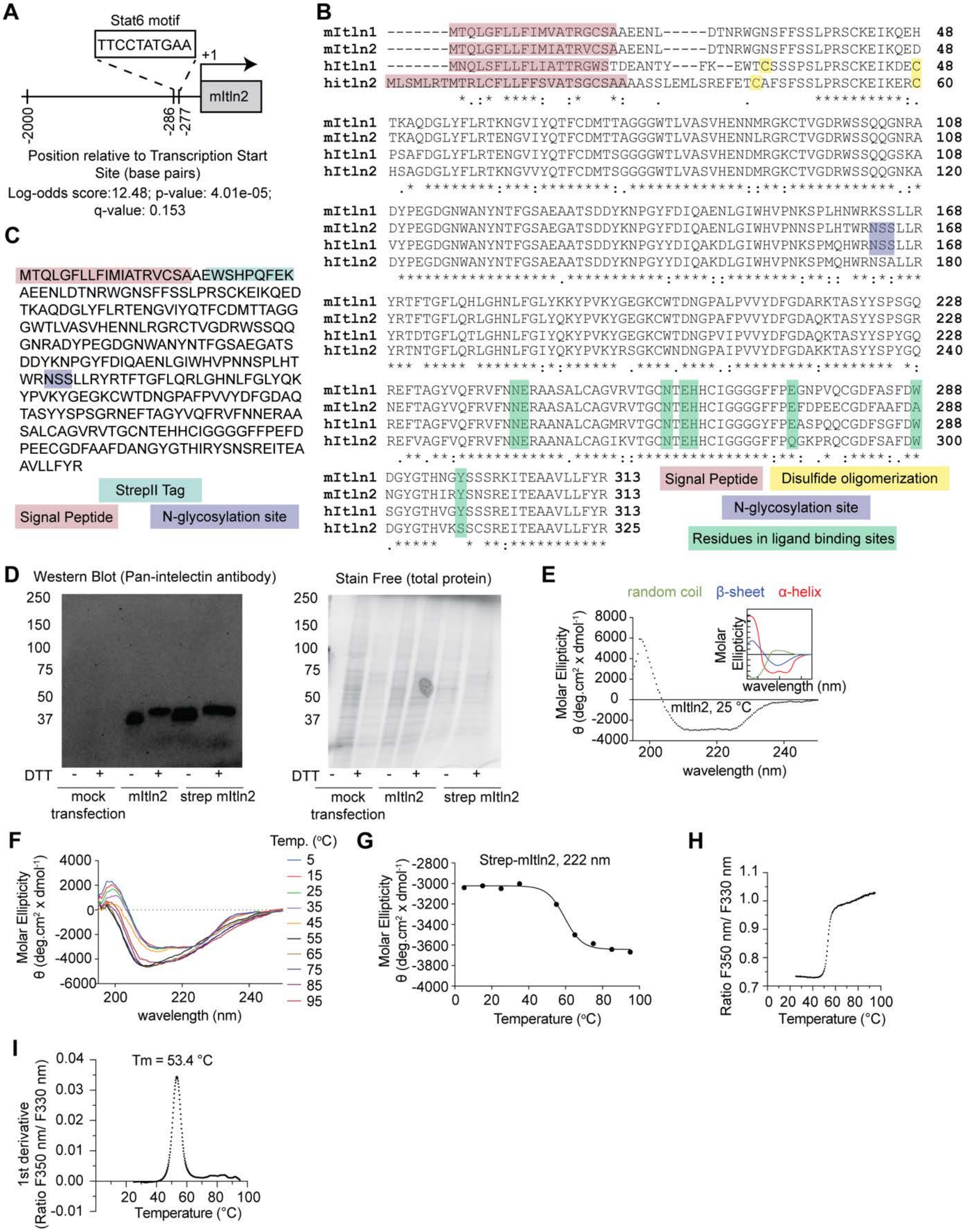
A
Stat6 motif
TTCCTATGAA
+1
mItln2
-2000
-286
-277
Position relative to Transcription Start Site (base pairs)
Log-odds score:12.48; p-value: 4.01e-05; q-value: 0.153
B
mItln1 -------MTQLGFLLFIMVATRGCSAAEENL-----DTNRWGNSFFSSLPRSCKEIKQEH 48
mItln2 -------MTQLGFLLFIMIATRVCSAAEENL-----DTNRWGNSFFSSLPRSCKEIKQED 48
hItln1 -------MNQLSFLLFLIATTRGWSTDEANTY---FK--EWTCSSSPSLPRSCKEIKDEC 48
hitln2 MLSMLRTMTRLCFLLFFSVATSGCSAAAASSLEMLSREFETCAFSFSSLPRSCKEIKERC 60
mItln1 TKAQDGLYFLRTKNGVIYQTFCDMTTAGGGWTLVASVHENNMRGKCTVGDRWSSQQGNRA 108
mItln2 TKAQDGLYFLRTENGVIYQTFCDMTTAGGGWTLVASVHENNLRGRCTVGDRWSSQQGNRA 108
hItln1 PSAFDGLYFLRTENGVIYQTFCDMTSGGGGWTLVASVHENDMRGKCTVGDRWSSQQGSKA 108
hItln2 HSAGDGLYFLRTKNGVVYQTFCDMTSGGGGWTLVASVHENDMRGKCTVGDRWSSQQGNKA 120
mItln1 DYPEGDGNWANYNTFGSAEAATSDDYKNPGYFDIQAENLGIWHVPNKSPLHNWRKSSLLR 168
mItln2 DYPEGDGNWANYNTFGSAEGATSDDYKNPGYFDIQAENLGIWHVPNNSPLHTWRNSSLLR 168
hItln1 VYPEGDGNWANYNTFGSAEAATSDDYKNPGYYDIQAKDLGIWHVPNKSPMQHWRNSSLLR 168
hItln2 DYPEGDGNWANYNTFGSAEAATSDDYKNPGYYDIQAKDLGIWHVPNKSPMQHWRNSALLR 180
mItln1 YRTFTGFLQHLGHNLFGLYKKYPVKYGEGKCWTDNGPALPVVYDFGDARKTASYYSPSGQ 228
mItln2 YRTFTGFLQRLGHNLFGLYQKYPVKYGEGKCWTDNGPAFPVVYDFGDAQKTASYYSPSGR 228
hItln1 YRTDTGFLQTLGHNLFGIYQKYPVKYGEGKCWTDNGPVIPVVYDFGDAQKTASYYSPYGQ 228
hItln2 YRTNTGFLQRLGHNLFGIYQKYPVKYRSGKCWNDNGPAIPVVYDFGDAKKTASYYSPYGQ 240
mItln1 REFTAGYVQFRVFNNERAASALCAGVRVTGCNTEHHCIGGGGFFPEGNPVQCGDFASFDW 288
mItln2 NEFTAGYVQFRVFNNERAASALCAGVRVTGCNTEHHCIGGGGFFPEFDPEECGDFAAFDA 288
hItln1 REFTAGFVQFRVFNNERAANALCAGMRVTGCNTEHHCIGGGGYFPEASPQQCGDFSGFDW 288
hItln2 REFVAGFVQFRVFNNERAANALCAGIKVTGCNTEHHCIGGGGFFPQGKPRQCGDFSAFDW 300
mItln1 DGYGTHNGYSSSRKITEAAVLLFYR 313
mItln2 NGYGTHIRYSNSREITEAAVLLFYR 313
hItln1 SGYGTHVGYSSSREITEAAVLLFYR 313
hItln2 DGYGTHVKSSCSREITEAAVLLFYR 325
Signal Peptide
Disulfide oligomerization
N-glycosylation site
Residues in ligand binding sites
C
MTQLGFLLFIMIATRVCSAAEWSHPQFEK
AEENLDTNRWGNSFFSSLPRSCKEIKQED
TKAQDGLYFLRTENGVIYQTFCDMTTAGG
GWTLVASVHENNLRGRCTVGDRWSSQQ
GNRADYPEGDGNWANYNTFGSAEGATS
DDYKNPGYFDIQAENLGIWHVPNNSPLHT
WRNSSLLRYRTFTGFLQRLGHNLFGLYQK
YPVKYGEGKCWTDNGPAFPVVYDFGDAQ
TASYYSPSGRNEFTAGYVQFRVFNNERAA
SALCAGVRVTGCNTEHHCIGGGGFFPEFD
PEECGDFAAFDANGYGTHIRYSNSREITEA
AVLLFYR
StrepII Tag
Signal Peptide
N-glycosylation site
D
Western Blot (Pan-intelectin antibody)
Stain Free (total protein)
250
150
100
75
50
37
DTT
mock transfection
mItln2
strep mItln2
E
random coil
β-sheet
α-helix
Molar Ellipticity θ (deg.cm² x dmol⁻¹)
mItln2, 25 °C
wavelength (nm)
F
Temp. (°C)
5
15
25
35
45
55
65
75
85
95
Molar Ellipticity θ (deg.cm² x dmol⁻¹)
wavelength (nm)
G
Strep-mItln2, 222 nm
Molar Ellipticity θ (deg.cm² x dmol⁻¹)
Temperature (°C)
H
Ratio F350 nm/ F330 nm
Temperature (°C)
I
Tm = 53.4 °C
1st derivative (Ratio F350 nm/ F330 nm)
Temperature (°C)

Supplementary Fig 1: Expression and biophysical characterization of recombinant mItln2.

(**A**) Putative Stat6-binding site in the promoter region of mItln2 as determined using FIMO analysis. The genomic position of the Stat6 motif, relative to the transcription start site, and the log-odds score (score for the match), *p*-value, and q-value (false-discovery rate) are indicated. (**B**) MUSCLE sequence alignment of mouse and human intelectins-1 and -2. Signal peptide (pink), N-glycosylation motifs (purple), and ligand binding residues (green) are highlighted. Cysteine residues needed for oligomerization of hItln1 but absent in mItln1 and mItln2 are indicated (yellow). Conserved, highly similar, and similar residues are represented by "*", ":", and "." respectively. (**C**) Sequence of StrepII tagged mItln2. Signal peptide (pink), StrepII-tag (cyan), and N-glycosylation motifs (purple) are highlighted. (**D**) Western blot (top) and corresponding stain-free gel (bottom) of the culture medium from HEK293T cells transfected with native mItln2 (no-tag) or StrepII-tagged mItln2 run under reducing and non-reducing conditions. Recombinant mItln2 was detected using a pan-intelectin antibody. (**E** and **F**) Circular dichroism spectra of mItln2 protein (500 µg/mL in PBS buffer) from 180 nm to 260 nm at different temperatures. Standard spectra of the basic secondary structures of a polypeptide chain (α-helix, β-sheet, and random coil) are embedded in (E). (**G**) Changes in ellipticity of mItln2 protein at 222 nm as a function of temperature, derived from data in (E). (**H** and **I**) Ratio of change in intrinsic fluorescence intensity at 330 nm and 350 nm (H) and its first derivative (I) as a function of temperature for recombinant mItln2, obtained from differential scanning fluorimetry. F, fluorescence. Results shown in (D), (E), (F), and (G) are representatives of two independent experiments. Results shown in (H) and (I) are representatives of three independent experiments. Source data are provided as source data file.

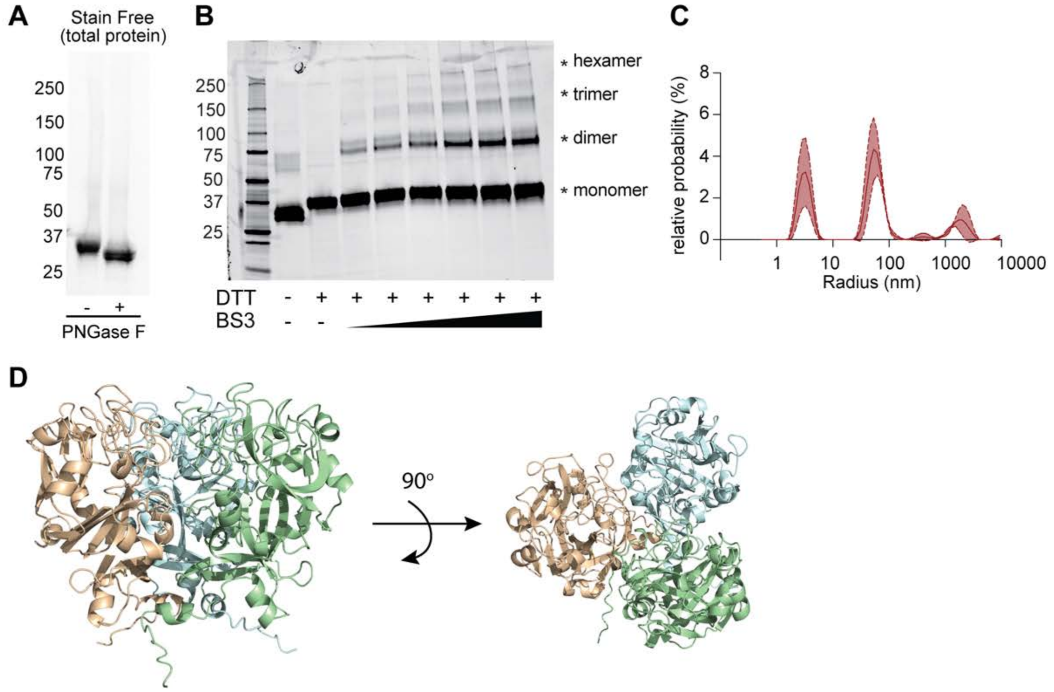

Supplementary Fig 2: Characterization of mItln2 glycosylation and oligomerization. (**A**) Stain-free gel of untreated and PNGase F-treated mItln2 proteins under reducing condition, demonstrating a decrease in molecular weight after removal of glycans. (**B**) SYPRO Ruby-stained SDS-PAGE of mItln2 proteins (1 mg/ mL) crosslinked with various concentrations of bis(sulfosuccinimidyl)suberate (BS3: 0.1 mM, 0.25 mM, 0.5 mM, 1.25 mM, 2.5 mM, and 5 mM). Samples were run under either reducing or non-reducing conditions as indicated. Molecular weight of monomeric mItln2 is 34 kDa, and different oligomers are indicated by *. (**C**) Relative distributions of particle sizes for mItln2 protein, obtained from dynamic light scattering. (**D)** Alphafold 3 predicted structure of mItln2 trimer. Individual monomers are displayed in wheat, green, and cyan. Results shown in (A), (B), and (C) are representatives of three independent experiments. Source data are provided as source data file.

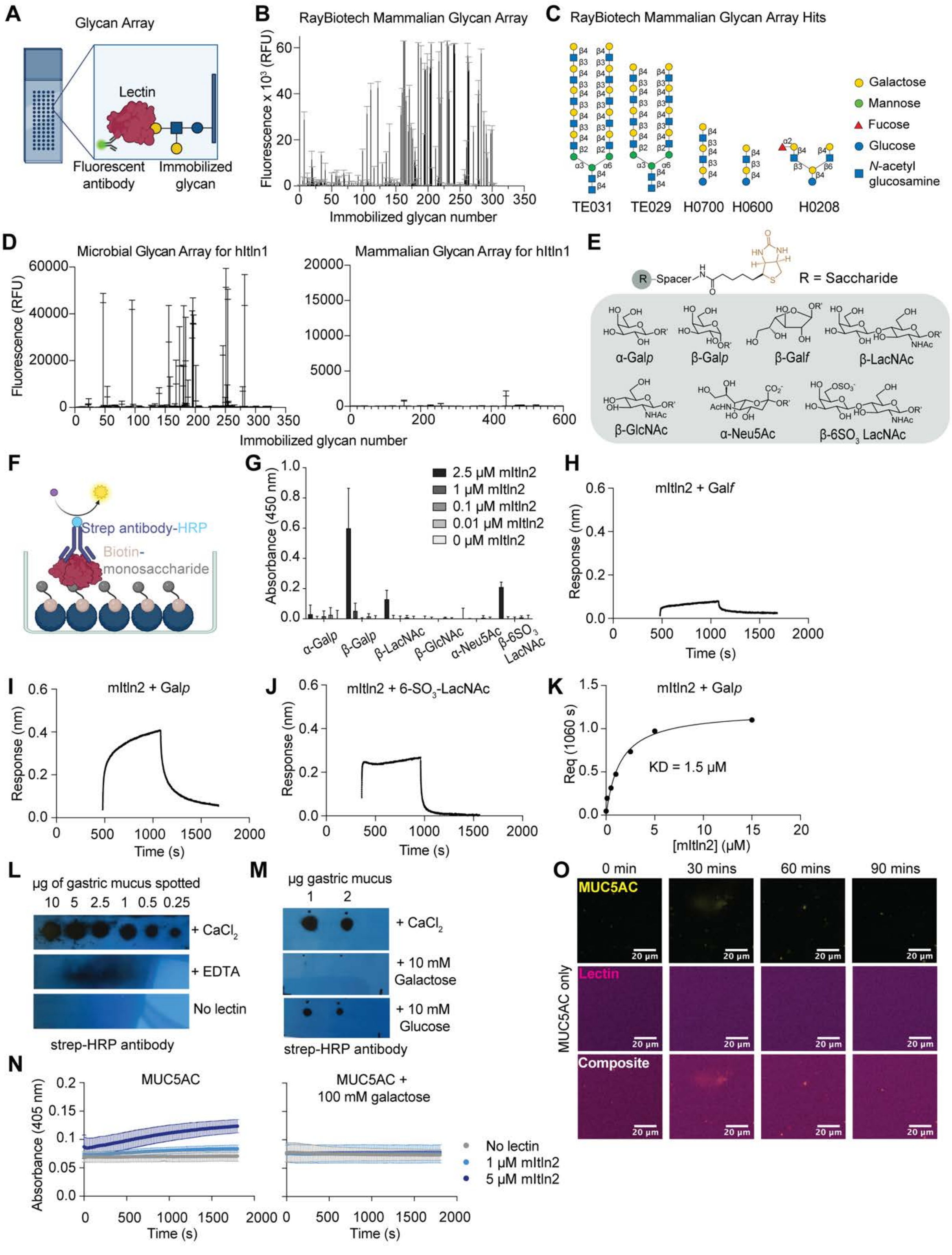
A
Glycan Array
Lectin
Fluorescent antibody
Immobilized glycan
B
RayBiotech Mammalian Glycan Array
Fluorescence x 10³ (RFU)
Immobilized glycan number
C
RayBiotech Mammalian Glycan Array Hits
TE031
TE029
H0700
H0600
H0208
Galactose
Mannose
Fucose
Glucose
N-acetyl glucosamine
D
Microbial Glycan Array for hItln1
Mammalian Glycan Array for hItln1
Fluorescence (RFU)
Immobilized glycan number
E
Spacer
R = Saccharide
α-Galp
β-Galp
β-Galf
β-LacNAc
β-GlcNAc
α-Neu5Ac
β-6SO3 LacNAc
F
Strep antibody-HRP
Biotin-monosaccharide
G
Absorbance (450 nm)
2.5 μM mItln2
1 μM mItln2
0.1 μM mItln2
0.01 μM mItln2
0 μM mItln2
α-Galp
β-Galp
β-LacNAc
β-GlcNAc
α-Neu5Ac
β-6SO3 LacNAc
H
mItln2 + Galf
Response (nm)
Time (s)
I
mItln2 + Galp
Response (nm)
Time (s)
J
mItln2 + 6-SO3-LacNAc
Response (nm)
Time (s)
K
mItln2 + Galp
Req (1060 s)
KD = 1.5 μM
[mItln2] (μM)
L
μg of gastric mucus spotted
10 5 2.5 1 0.5 0.25
+ CaCl2
+ EDTA
No lectin
strep-HRP antibody
M
μg gastric mucus
1 2
+ CaCl2
+ 10 mM Galactose
+ 10 mM Glucose
strep-HRP antibody
N
MUC5AC
MUC5AC + 100 mM galactose
Absorbance (405 nm)
Time (s)
No lectin
1 μM mItln2
5 μM mItln2
O
0 min
30 mins
60 mins
90 mins
MUC5AC
Lectin
Composite
MUC5AC only
20 μm

Supplementary Fig 3: Carbohydrate binding specificity of recombinant mItln2.
(A) Schematic of glycan array analysis. Recombinant lectins are allowed to bind synthetic and natural glycans immobilized on a glass slide. Lectin binding is detected with a fluorescent antibody against the lectin. Created in BioRender. Lab01, K. (2025) https://BioRender.com/8hzu1xa. (B) Binding profile of recombinant mItln2 (25 μg/mL) to mammalian glycan microarrays from RayBiotech. Data are shown as mean ± SD (n = 4 technical replicates). (C) Graphical representations of top five glycan hits for mItln2 on the RayBiotech mammalian glycan arrays. Complete microarray data are found in Supplemental Table 2. (D) Binding profiles of recombinant hItln1 (50μg/mL) to microbial (left) and mammalian (right) glycan microarrays from NCFG (*12*). Data are shown as mean ± SD (n = 4 technical replicates). (E) Structures of biotin-functionalized carbohydrates used in this study. (F) Schematic of enzyme-linked lectin assay (ELLA). Biotinylated carbohydrates are anchored to surface via streptavidin, and bound lectin is detected using a Strep-HRP antibody and colorimetric HRP substrate. Created in BioRender. Lab01, K. (2025) https://BioRender.com/l10bj0i. (G) ELLA assay for mItln2 binding to immobilized carbohydrates at varying mItln2 concentrations. Data are shown as mean ± SD (n= 3 technical replicates). (H - J) BLI trace of mItln2 binding to immobilized biotinylated-β-Gal*f* (H), biotinylated-β-Gal*p* (I), and biotinylated-6SO_3 LacNAc (J). Binding was tested with 1.5 μM mItln2 for β-Gal*f* and β-Gal*p*, and 5 μM mItln2 for 6SO_3 LacNAc. Data were normalized by background subtraction using biotin-loaded streptavidin. (K) BLI response of mItln2 binding to immobilized biotinylated-Gal*p* at 1060 s (depicted in Fig. 2D) was plotted against mItln2 concentration and fitted to a single site binding equation to determine KD. (L and M) Dot blot analysis for different amounts of gastric mucus on nitrocellulose membrane was performed with 0.5 μM mItln2, either in the presence of Ca^{2+} or EDTA. Controls included no lectin (L) and treatment with 10 mM carbohydrates (galactose or glucose) (M). Binding of mItln2 was detected using a Strep-HRP antibody. (N) Spectroscopic assay for crosslinking of 0.05% (w/v) MUC5AC with varying amounts of mItln2 (with and without 100 mM galactose), measured by the increase in absorbance at 405 nm. Data are shown as mean ± SD (n = 3 technical replicates). (O) Time-lapse images of 0.01% (w/v) fluorescently labeled MUC5AC (yellow) without mItln2 (magenta) treatment as a control for experiment depicted in Figure 2H. Binding of mItln2 was detected with Strep antibody. Scale bars, 20 μm. Results in (J), (L), (M), (N), and (O) are representative of two independent experiments. Results in (G), (H), (I), and (K) are representative of three independent experiments. RFU, Relative Fluorescence Unit. Source data are provided as source data file.

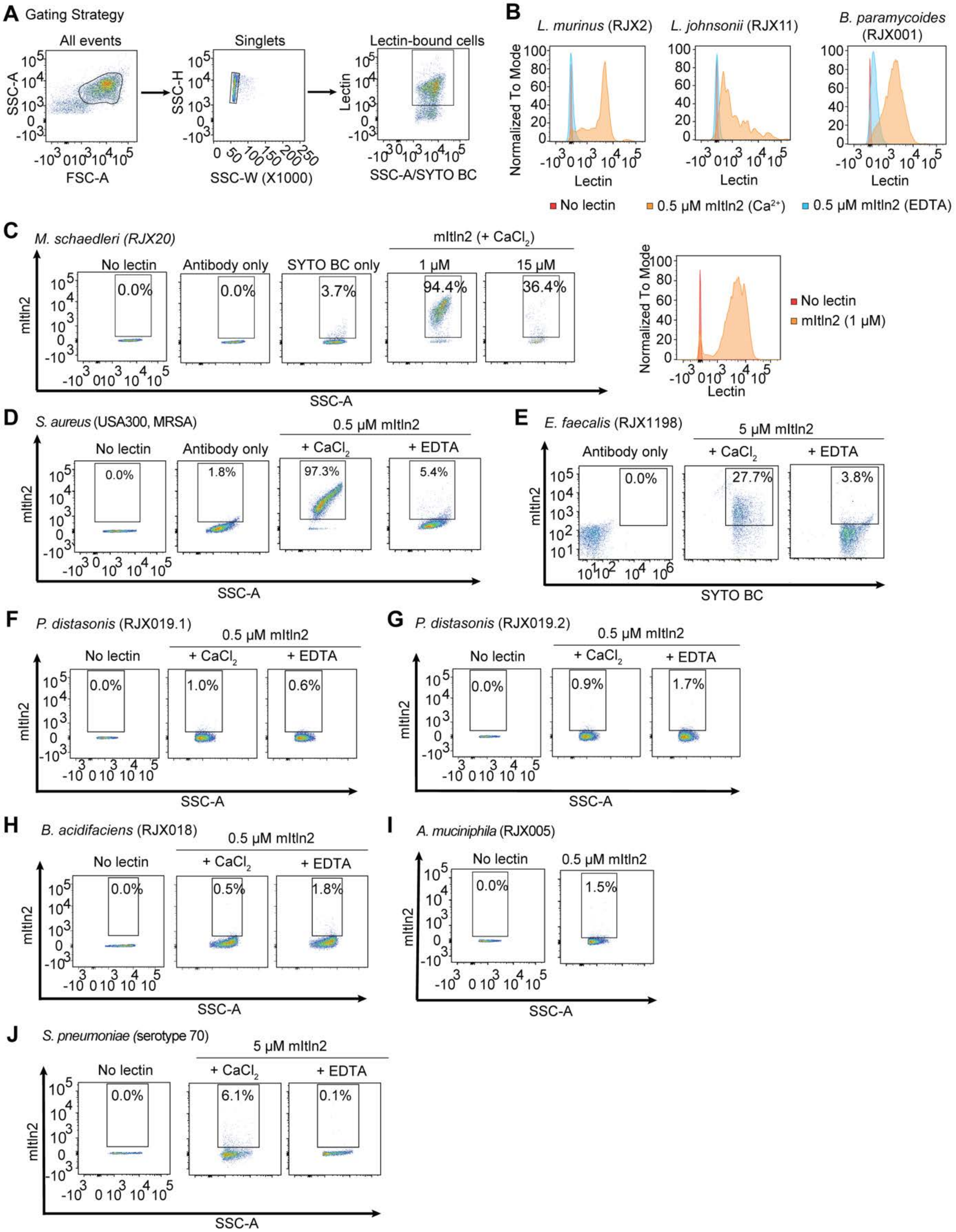
A Gating Strategy
All events
Singlets
Lectin-bound cells
SSC-A
FSC-A
SSC-H
SSC-W (X1000)
Lectin
SSC-A/SYTO BC
B
L. murinus (RJX2)
L. johnsonii (RJX11)
B. paramycoides (RJX001)
Normalized To Mode
Lectin
No lectin
0.5 μM mItln2 (Ca2+)
0.5 μM mItln2 (EDTA)
C
M. schaedleri (RJX20)
mItln2 (+ CaCl2)
No lectin
Antibody only
SYTO BC only
1 μM
15 μM
0.0%
0.0%
3.7%
94.4%
36.4%
mItln2
SSC-A
No lectin
mItln2 (1 μM)
D
S. aureus (USA300, MRSA)
0.5 μM mItln2
No lectin
Antibody only
+ CaCl2
+ EDTA
0.0%
1.8%
97.3%
5.4%
E
E. faecalis (RJX1198)
5 μM mItln2
Antibody only
+ CaCl2
+ EDTA
0.0%
27.7%
3.8%
SYTO BC
F
P. distasonis (RJX019.1)
0.5 μM mItln2
No lectin
+ CaCl2
+ EDTA
0.0%
1.0%
0.6%
G
P. distasonis (RJX019.2)
0.5 μM mItln2
No lectin
+ CaCl2
+ EDTA
0.0%
0.9%
1.7%
H
B. acidifaciens (RJX018)
0.5 μM mItln2
No lectin
+ CaCl2
+ EDTA
0.0%
0.5%
1.8%
I
A. muciniphila (RJX005)
No lectin
0.5 μM mItln2
0.0%
1.5%
J
S. pneumoniae (serotype 70)
5 μM mItln2
No lectin
+ CaCl2
+ EDTA
0.0%
6.1%
0.1%

Supplementary Fig 4: Binding profiles of mItln2 to different gram-positive and gram-negative microbial isolates.

(A) Flow cytometry gating strategy used to assess lectin binding to microbes.

(B) Flow cytometry histogram plots showing mItln2 binding to *L. murinus* RJX2, *L. johnsonii* RJX11, and *B. paramycoides* RJX001 under Ca^{2+} or EDTA conditions. mItln2 was detected by Strep antibody. Unstained samples (no lectin) served as a control. (C – J) Flow cytometry analysis of mItln2 binding to *M. schaedleri* RJX020 (C), *S. aureus* MRSA (D), *E. faecalis* RJX1198 (E), *P. distasonis* RJX019.1 (F), *P. distasonis* RJX019.2 (G), *B. acidifaciens* RJX018 (H), *A. muciniphilia* RJX005 (I), and *S. pneumoniae* serotype 70 (J). Dot plots show lectin binding (anti-Strep DY549) vs. SSC or nucleic acid stain (SYTO BC). Histograms display cell counts as a percent of the maximum signal against lectin binding in different conditions. Unstained samples (no lectin) or samples treated with SYTO BC or Strep antibody alone served as controls. Data in (B), (C), (F), (G), (H), (I), and (J) are representative of two independent experiments. Data in (D), (E) are representative of three independent experiments.

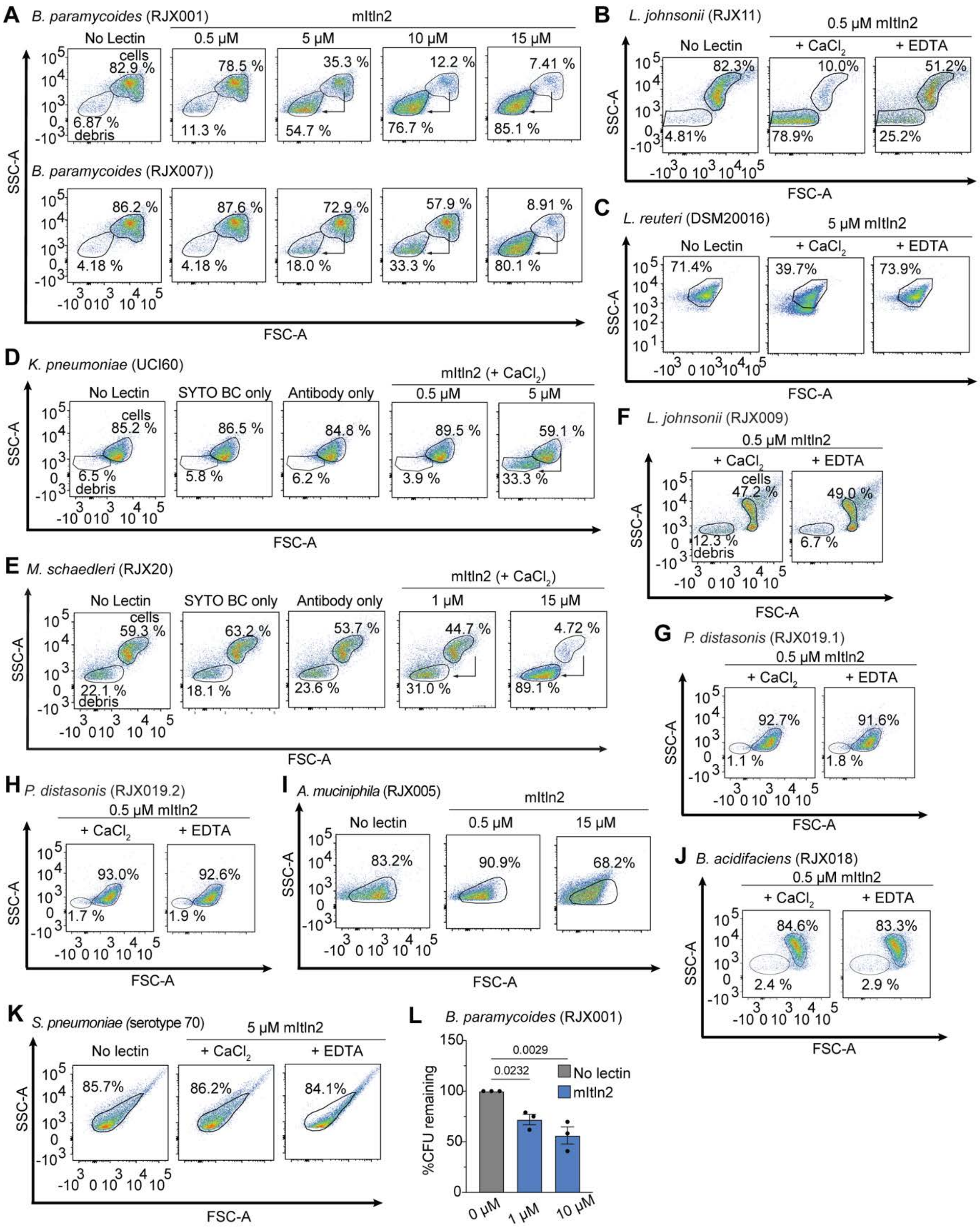
A B. paramycoides (RJX001)
mItln2
No Lectin
0.5 µM
5 µM
10 µM
15 µM
cells
82.9 %
78.5 %
35.3 %
12.2 %
7.41 %
6.87 %
debris
11.3 %
54.7 %
76.7 %
85.1 %
B. paramycoides (RJX007))
86.2 %
87.6 %
72.9 %
57.9 %
8.91 %
4.18 %
4.18 %
18.0 %
33.3 %
80.1 %
SSC-A
FSC-A
B L. johnsonii (RJX11)
0.5 µM mItln2
No Lectin
+ CaCl2
+ EDTA
82.3%
10.0%
51.2%
4.81%
78.9%
25.2%
C L. reuteri (DSM20016)
5 µM mItln2
No Lectin
+ CaCl2
+ EDTA
71.4%
39.7%
73.9%
D K. pneumoniae (UCI60)
mItln2 (+ CaCl2)
No Lectin
SYTO BC only
Antibody only
0.5 µM
5 µM
cells
85.2 %
86.5 %
84.8 %
89.5 %
59.1 %
6.5 %
debris
5.8 %
6.2 %
3.9 %
33.3 %
E M. schaedleri (RJX20)
mItln2 (+ CaCl2)
No Lectin
SYTO BC only
Antibody only
1 µM
15 µM
cells
59.3 %
63.2 %
53.7 %
44.7 %
4.72 %
22.1 %
debris
18.1 %
23.6 %
31.0 %
89.1 %
F L. johnsonii (RJX009)
0.5 µM mItln2
+ CaCl2
+ EDTA
cells
47.2 %
49.0 %
12.3 %
debris
6.7 %
G P. distasonis (RJX019.1)
0.5 µM mItln2
+ CaCl2
+ EDTA
92.7%
91.6%
1.1 %
1.8 %
H P. distasonis (RJX019.2)
0.5 µM mItln2
+ CaCl2
+ EDTA
93.0%
92.6%
1.7 %
1.9 %
I A. muciniphila (RJX005)
mItln2
No lectin
0.5 µM
15 µM
83.2%
90.9%
68.2%
J B. acidifaciens (RJX018)
0.5 µM mItln2
+ CaCl2
+ EDTA
84.6%
83.3%
2.4 %
2.9 %
K S. pneumoniae (serotype 70)
5 µM mItln2
No lectin
+ CaCl2
+ EDTA
85.7%
86.2%
84.1%
L B. paramycoides (RJX001)
%CFU remaining
0.0029
0.0232
No lectin
mItln2
0 µM
1 µM
10 µM

Supplementary Fig. 5: Assessment of cell integrity and lectin activity in mItln2-binding and non-binding microbes.
(**A** – **K**) Flow cytometry analysis of cell integrity of *B. paramycoides* RJX001 and RJX007 (A), *L. johnsonii* RJX11 (B), *L. reuteri* DSM20016 (C), *K. pneumoniae* UCI60 (D), *M. schaedleri* RJX20 (E), *L. johnonii* RJX009 (F), *P. distasonis* RJX019.1 (G), *P. distasonis* RJX019.2 (H), *A. muciniphilia* RJX005 (I), *B. acidifaciens* RJX018 (J), and *S. pneumoniae* serotype 70 (K) following treatment with mItln2 at different concentrations. Dot plots show SSC vs FSC. Regions representing debris and bacterial cells are indicated. Unstained samples (no lectin) or samples treated with SYTO BC or Strep antibody alone served as controls. Data in (A), (B), (C), (D), (E), (F), (G), (H), (I), (J), and (K) are representative of two independent experiments. (**L**) Quantification of viable *B. paramycoides* RJX001 by dilution plating after incubation with various concentrations of mItln2 for 4 hours. Data show mean ± SD (n = 3 independent experiments; one-way ANOVA followed by Dunnett's multiple comparisons test). Source data are provided as source data file.

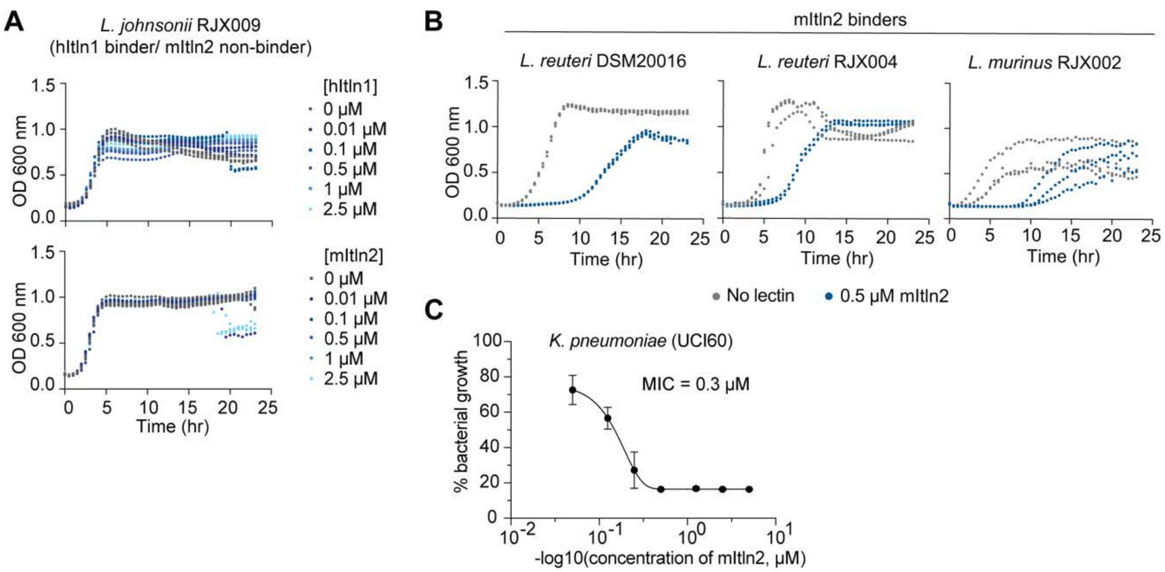

Supplementary Fig. 6: Measurement of microbial growth after mItln2 treatment.
(**A** and **B**) Recovery assay, assessing microbial growth (by OD_{600} measurements), after lectin treatment to different *Lactobacilli* bacteria (n = 3 technical replicates). Untreated microbes served as controls. Data in (A) and (B) are representative of two independent experiments. (**C**) Percentage of *K. pneumoniae* (UCI60) growth, calculated from OD_{600} measurements at 960 mins in Fig. 4F, in the presence of varying concentration of mItln2. The MIC value was determined by fitting the % growth data to Gompertz functions. Data represent mean value from n = 3 independent experiments in Fig. 4H. Source data are provided as source data file.

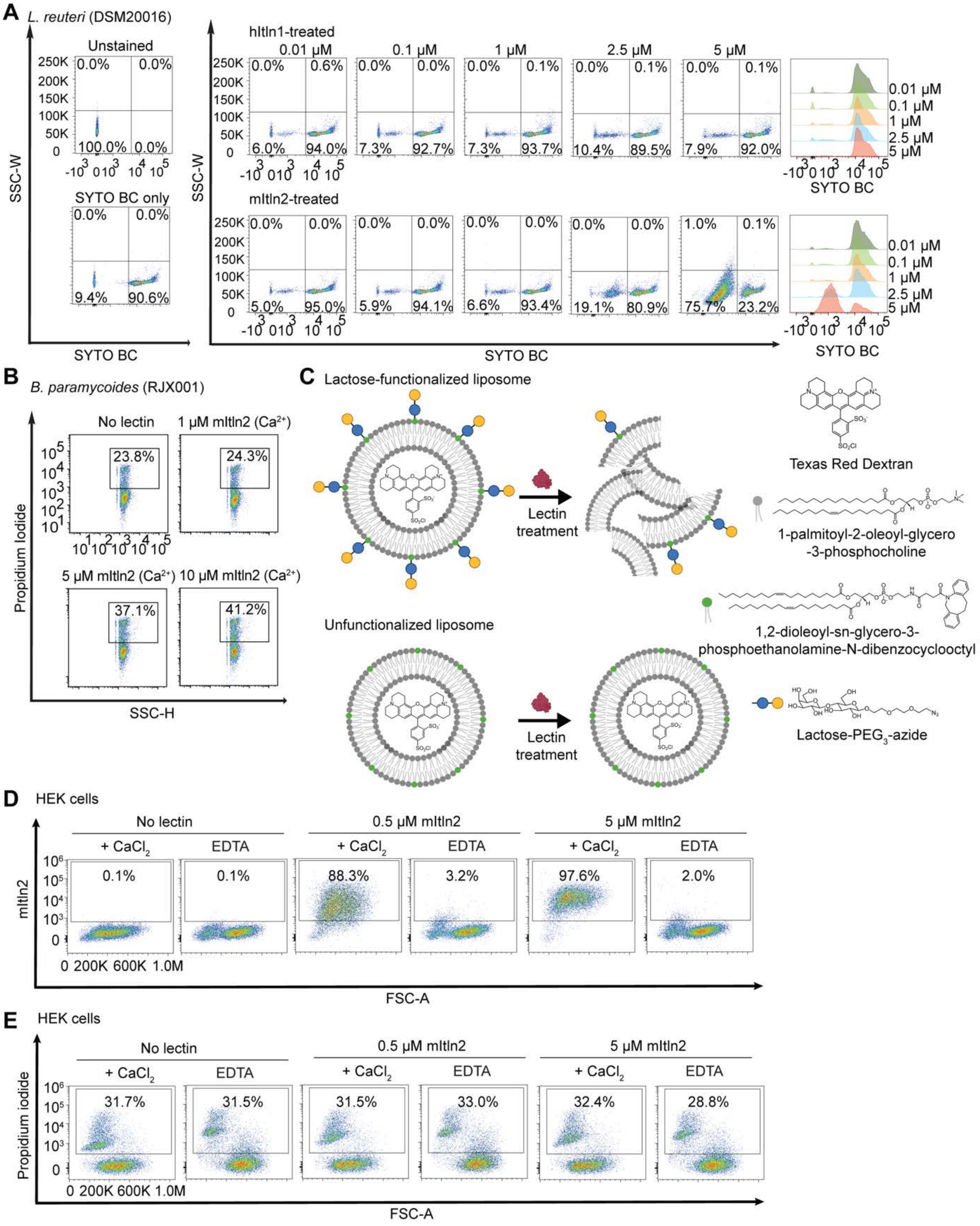
A
L. reuteri (DSM20016)
Unstained
SSC-W
SYTO BC only
SYTO BC
hItln1-treated
0.01 μM
0.1 μM
1 μM
2.5 μM
5 μM
mItln2-treated
B
B. paramycoides (RJX001)
No lectin
1 μM mItln2 (Ca2+)
5 μM mItln2 (Ca2+)
10 μM mItln2 (Ca2+)
Propidium iodide
SSC-H
C
Lactose-functionalized liposome
Lectin treatment
Texas Red Dextran
1-palmitoyl-2-oleoyl-glycero-3-phosphocholine
1,2-dioleoyl-sn-glycero-3-phosphoethanolamine-N-dibenzocyclooctyl
Unfunctionalized liposome
Lactose-PEG3-azide
D
HEK cells
No lectin
0.5 μM mItln2
5 μM mItln2
+ CaCl2
EDTA
mItln2
FSC-A
E
HEK cells
Propidium iodide
FSC-A

Supplementary Fig. 7: Assessment of microbial and mammalian cell viability after mItln2 treatment.

(**A**) Flow cytometry assessment of cell integrity in *L. reuteri* DSM20016 after treatment with mItln2 at different concentrations, using SYTO BC labeling. Loss of SYTO signal indicates compromised cells. (**B**) Flow cytometry assessment of cell integrity in *B. paramycoides* RJX001 after treatment with mItln2 at different concentrations, using propidium iodide labeling. Gain of propidium iodide signal indicates compromised cells. (**C**) Schematic of liposome disruption assay. Liposomes (200 nm diameter) composed of palmitoyl oleoyl phosphatidylcholine (POPC) with 10 mol% cholesterol for membrane fluidity and 10 mol% 1,2-dioleoyl-sn-glycero-3-phosphoethanolamine-N-dibenzocyclooctyl (18:1 DBCO PE) for glycan attachment were used. The liposomes were encapsulated with Texas red-labeled dextran (m.w. 3000 Da) and functionalized with lactose-PEG_3-azide via strain-promoted azido-alkynyl cycloaddition. Addition of mItln2 to the lactose-functionalized liposomes (top) led to a concentration-dependent increase in absorbance at 405 nm and dye release, indicating lipid bilayer disruption. Liposomes without glycan functionalization (bottom) served as controls and showed no membrane disruption, confirming the glycan specificity of the effect. Created in BioRender. Lab01, K. (2025) https://BioRender.com/sjq9lj5. (**D**) Flow cytometry analysis of mItln2 binding to HEK293 cells under Ca^{2+} or EDTA conditions. mItln2 was detected by Strep antibody. Dot plots show lectin binding (anti-Strep DY549) vs. FSC. (**E**) Flow cytometry assessment of cell integrity in HEK293 cells after treatment with mItln2 at different concentrations, using propidium iodide labeling. Gain of propidium iodide signal indicates increased membrane permeability of cells. Data in (A) and (B) are representative of three independent experiments. Data in (D) and (E) are representative of two independent experiments.

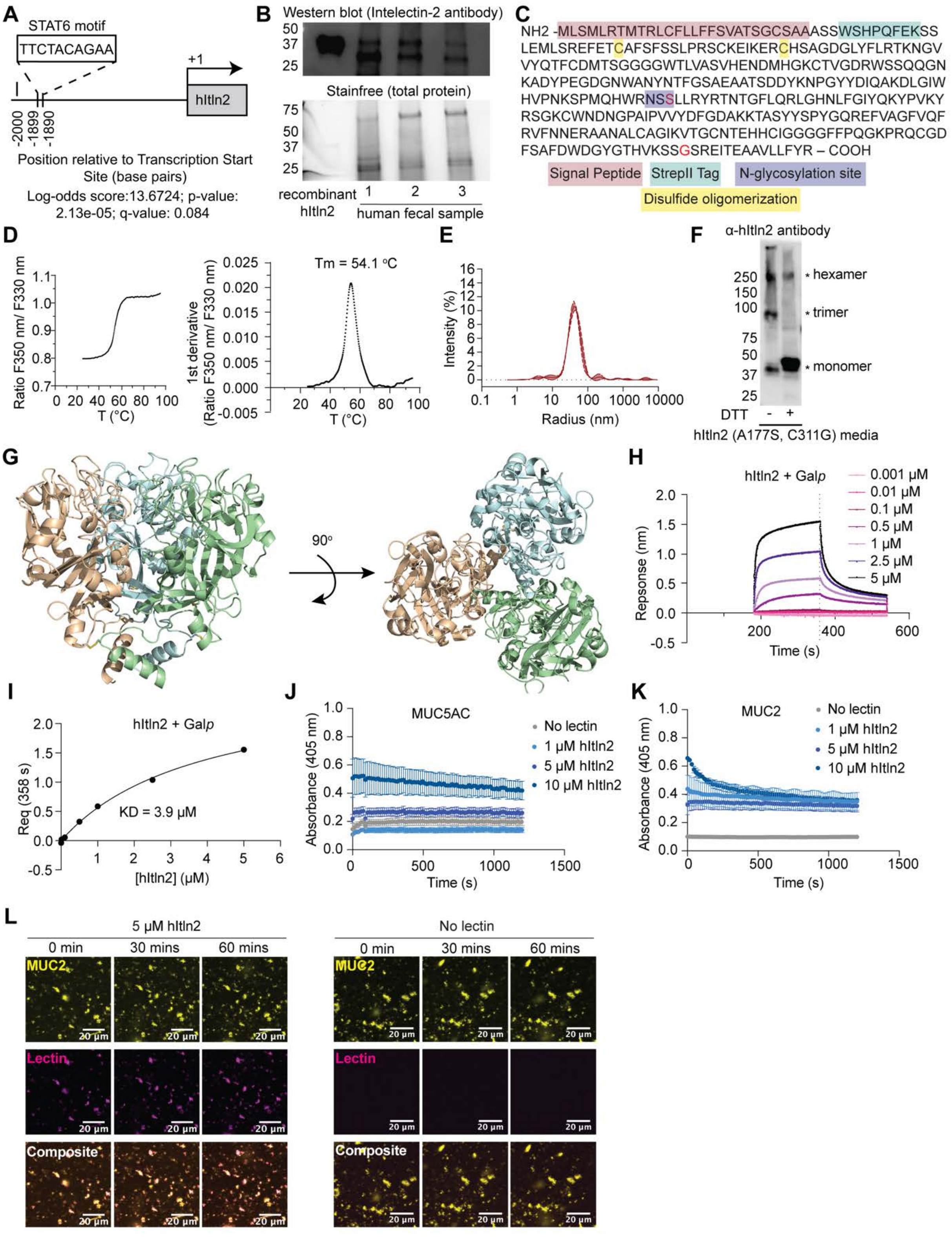
A
STAT6 motif
TTCTACAGAA
+1
hItln2
-2000
-1899
-1890
Position relative to Transcription Start Site (base pairs)
Log-odds score:13.6724; p-value: 2.13e-05; q-value: 0.084
B
Western blot (Intelectin-2 antibody)
Stainfree (total protein)
recombinant hItln2
1 2 3
human fecal sample
C
NH2 -MLSMLRTMTRLCFLLFFSVATSGCSAAASSWSHPQFEKSS
LEMLSREFETCAFSFSSLPRSCKEIKERCHSAGDGLYFLRTKNGV
VYQTFCDMTSGGGGWTLVASVHENDMHGKCTVGDRWSSQQGN
KADYPEGDGNWANYNTFGSAEAATSDDYKNPGYYDIQAKDLGIW
HVPNKSPMQHWRNSSLLRYRTNTGFLQRLGHNLFGIYQKYPVKY
RSGKCWNDNGPAIPVVYDFGDAKKTASYYSPYGQREFVAGFVQF
RVFNNERAANALCAGIKVTGCNTEHHCIGGGGFFPQGKPRQCGD
FSAFDWDGYGTHVKSSGSREITEAAVLLFYR – COOH
Signal Peptide
StrepII Tag
N-glycosylation site
Disulfide oligomerization
D
Ratio F350 nm/ F330 nm
T (°C)
Tm = 54.1 °C
1st derivative (Ratio F350 nm/ F330 nm)
E
Intensity (%)
Radius (nm)
F
α-hItln2 antibody
hexamer
trimer
monomer
DTT - +
hItln2 (A177S, C311G) media
G
90°
H
hItln2 + Galp
0.001 μM
0.01 μM
0.1 μM
0.5 μM
1 μM
2.5 μM
5 μM
Response (nm)
Time (s)
I
hItln2 + Galp
Req (358 s)
KD = 3.9 μM
[hItln2] (μM)
J
MUC5AC
No lectin
1 μM hItln2
5 μM hItln2
10 μM hItln2
Absorbance (405 nm)
Time (s)
K
MUC2
No lectin
1 μM hItln2
5 μM hItln2
10 μM hItln2
Absorbance (405 nm)
Time (s)
L
5 μM hItln2
No lectin
0 min
30 mins
60 mins
MUC2
Lectin
Composite
20 μm

Supplementary Fig. 8: Expression and characterization of recombinant hItln2.
(**A**) Putative STAT6-binding site in the promoter region of hItln2 as determined using FIMO analysis. The genomic position of the STAT6 motif, relative to the transcription start site, and the log-odds score (score for the match), *p*-value, and q-value (false-discovery rate) are indicated. (**B**) Western blot (top) and stain-free gel (bottom) of fecal samples from three individuals under reducing conditions. In Western blot, hItln2 was detected by an intelectin-2 specific antibody. Recombinant StrepII-tagged hItln2 was used as a control. (**C**) Amino acid sequences of StrepII-tagged hItln2, with the signal peptide (pink), StrepII-tag (cyan), cysteine residues (needed for oligomerization, yellow), and N-glycosylation motifs (purple) highlighted. Mutations (A177S and C311G) introduced to optimize recombinant hItln2 expression are labeled in red. (**D**) Ratio of change in intrinsic fluorescence intensity at 330 nm and 350 nm (left) and its first derivative (right) as a function of temperature for recombinant hItln2, obtained from differential scanning fluorimetry. (**E**) Relative distribution of particle sizes for hItln2 protein, as measured by dynamic light scattering. (**F**) Western blot of hItln2 protein under reducing or non-reducing conditions, visualized with anti-intelectin-2 antibody. Molecular weight of monomeric hItln2 is 34 kDa, and different oligomers are indicated by *. (**G**) AlphaFold 3 predicted trimeric structure for hItln2. Conserved cysteines form covalent linkages between individual monomers shown in wheat, green, and cyan. (**H**) BLI trace of hItln2 binding to immobilized biotinylated-β-Gal*p* at varying concentrations. Data were normalized by background subtraction, using biotin-loaded streptavidin. (**I**) BLI response of hItln2 binding to immobilized biotinylated-Gal*p* at 358 seconds, plotted against hItln2 concentration and fitted to a single-site binding equation, yielding a KD of 3.9 µM. (**J** and **K**) Spectroscopic assay measuring crosslinking of 0.05% (w/v) MUC5AC (J) and MUC2 (K) by hItln2, indicated by the increase in absorbance at 405 nm. Data are shown as mean ± SD (n = 3 technical replicates). (**L**) Time-lapse images of 0.01% (w/v) fluorescently labeled MUC2 (yellow) treated with 5 µM hItln2 (magenta). Binding of hItln2 was detected with Strep antibody. MUC2 without lectin treatment was used as a control. Scale bars, 20 µm. Results in (B), (D), (E), and (F) are representative of three independent experiments. Results in (H), (I), (J), (K), and (L) are representative of two independent experiments. Source data are found in the source data file.

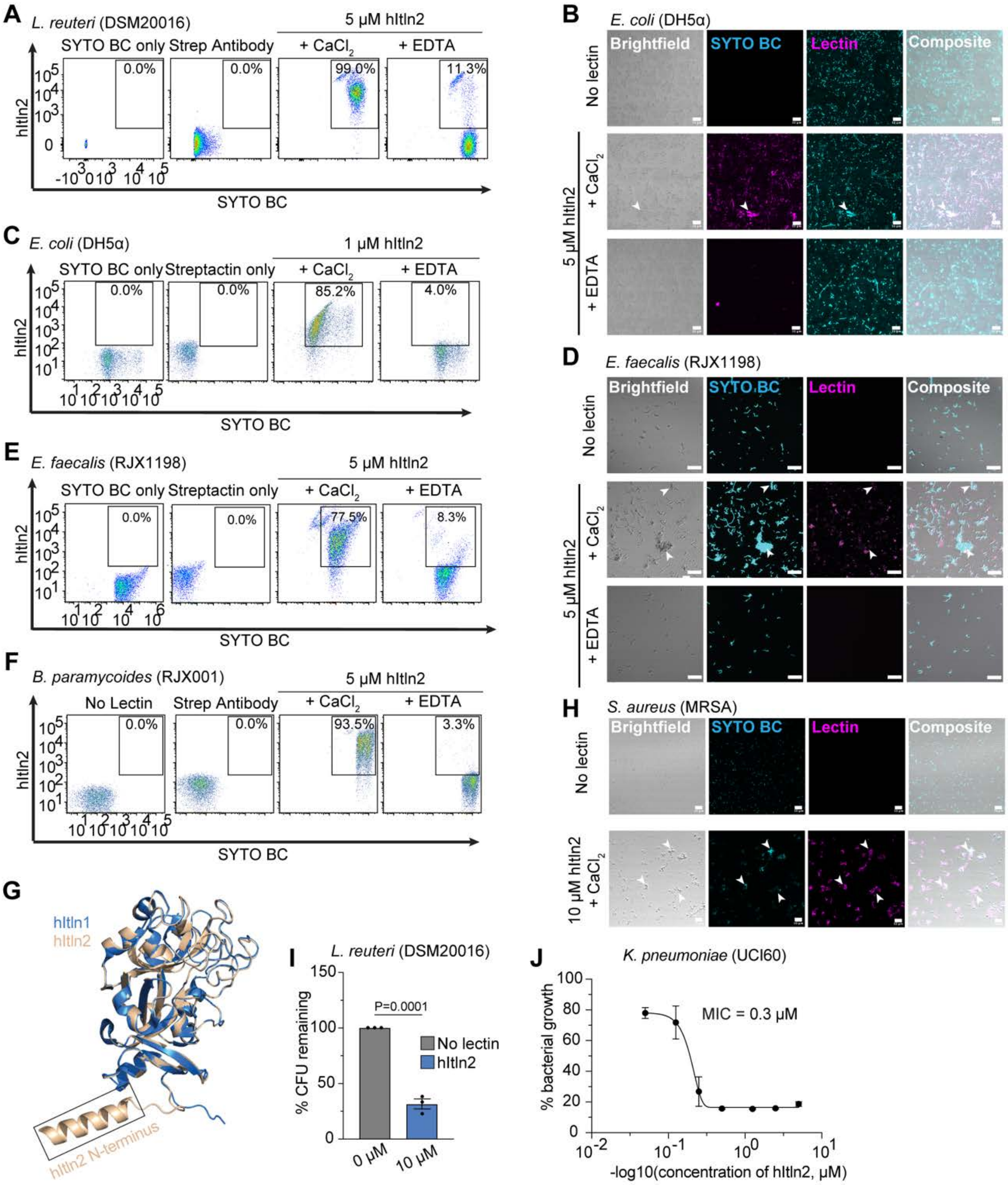
A
L. reuteri (DSM20016)
5 μM hItln2
SYTO BC only
Strep Antibody
+ CaCl2
+ EDTA
0.0%
0.0%
99.0%
11.3%
hItln2
SYTO BC
B
E. coli (DH5α)
Brightfield
SYTO BC
Lectin
Composite
No lectin
5 μM hItln2
+ CaCl2
+ EDTA
C
E. coli (DH5α)
1 μM hItln2
SYTO BC only
Streptactin only
+ CaCl2
+ EDTA
0.0%
0.0%
85.2%
4.0%
D
E. faecalis (RJX1198)
E
E. faecalis (RJX1198)
5 μM hItln2
SYTO BC only
Streptactin only
+ CaCl2
+ EDTA
0.0%
0.0%
77.5%
8.3%
F
B. paramycoides (RJX001)
5 μM hItln2
No Lectin
Strep Antibody
+ CaCl2
+ EDTA
0.0%
0.0%
93.5%
3.3%
H
S. aureus (MRSA)
10 μM hItln2
+ CaCl2
G
hItln1
hItln2
hItln2 N-terminus
I
L. reuteri (DSM20016)
% CFU remaining
P=0.0001
No lectin
hItln2
0 μM
10 μM
J
K. pneumoniae (UCI60)
% bacterial growth
MIC = 0.3 μM
-log10(concentration of hItln2, μM)

Supplementary Fig. 9: Binding profiles of hItln2 to different microbial cells.
(**A**) Flow cytometry analysis of hItln2 binding to *L. reuteri* DSM20016 under Ca^{2+} or EDTA conditions, detected by StrepII antibody. Dot plots showing lectin binding are displayed as anti-Strep DY549 vs. SYTO BC. (**B** and **C**) Microscopy images (B) and flow cytometry analysis (C) of hItln2 binding to *E. coli* DH5α under Ca^{2+} or EDTA conditions. hItln2 was detected by Streptactin. Scale bars, 10 µm. (**D** and **E**) Microscopy images (D) and flow cytometry analysis (E) of hItln2 binding to *E. faecalis* RJX1198 under Ca^{2+} or EDTA conditions. hItln2 was detected by Streptactin. Scale bars, 20 µm. (**F**) Flow cytometry analysis of hItln2 binding to *B. paramycoides* RJX1001 under Ca^{2+} or EDTA conditions, detected by StrepII antibody. Dot plots showing lectin binding are displayed as anti-Strep DY549 vs. SYTO BC. **(G)** Alignment of hItln1-monomer (blue, PDB ID 4WMY) and hItln2-monomer (wheat, predicted model), highlighting conserved protein structures. The lengthened N-terminal sequence in hItn2, predicted to form an alpha helix, is labeled. **(H)** Images of *S. aureus* MRSA stained with hItln2 (magenta) and counterstained with SYTO BC (teal). hItln2 was detected by StrepII antibody. Scale bars, 10 µm. (**I**) Quantification of viable *L. reuteri* DSM20016 by dilution plating after 4-hours incubation with various concentrations of hItln2. Data show mean ± SEM (n = 3 independent experiments; unpaired two-tailed t-test). (**J**) Percentage of *K. pneumoniae* (UCI60) growth, calculated from OD_{600} measurements at 960 mins in Fig. 6G, in the presence of varying concentration of hItln2. MIC value was determined by fitting the % growth data to Gompertz functions. Data represent mean value from n = 3 independent experiments in Fig. 6G. Treatment with SYTO BC and Strep antibody or Streptactin (without lectin) served as a control in (B), (D), and (H). Unstained samples (no lectin) or those treated with SYTO BC, Strep antibody, or Streptactin alone served as controls in (A), (C), (E), and (F). Examples of microbial agglutination are marked with white arrowheads in (B), (D), and (H). Results in (A), (B), and (D) are representative of two independent experiments. Result in (C), (E), (F), and (H) are representative of three independent experiments. Source data are found in the source data file.

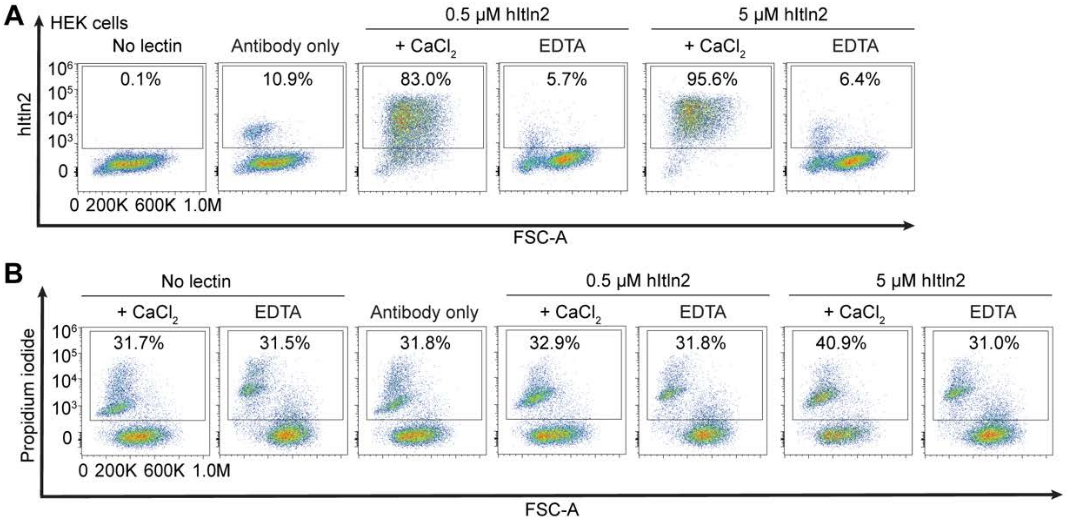

Supplementary Fig. 10: Binding profiles of hItln2 to mammalian cells.
(**A**) Flow cytometry analysis of hItln2 binding to HEK293 cells under Ca^{2+} or EDTA conditions. hItln2 was detected by Strep antibody. Dot plots show lectin binding (anti-Strep DY549) vs. FSC. (**B**) Flow cytometry assessment of cell integrity in HEK293 cells after treatment with hItln2, using propidum iodide labeling. Gain of propidium iodide signal indicates compromised cells. Treatment with Strep antibody (without lectin) served as a control. Results in (A) and (B) are representative of two independent experiments.

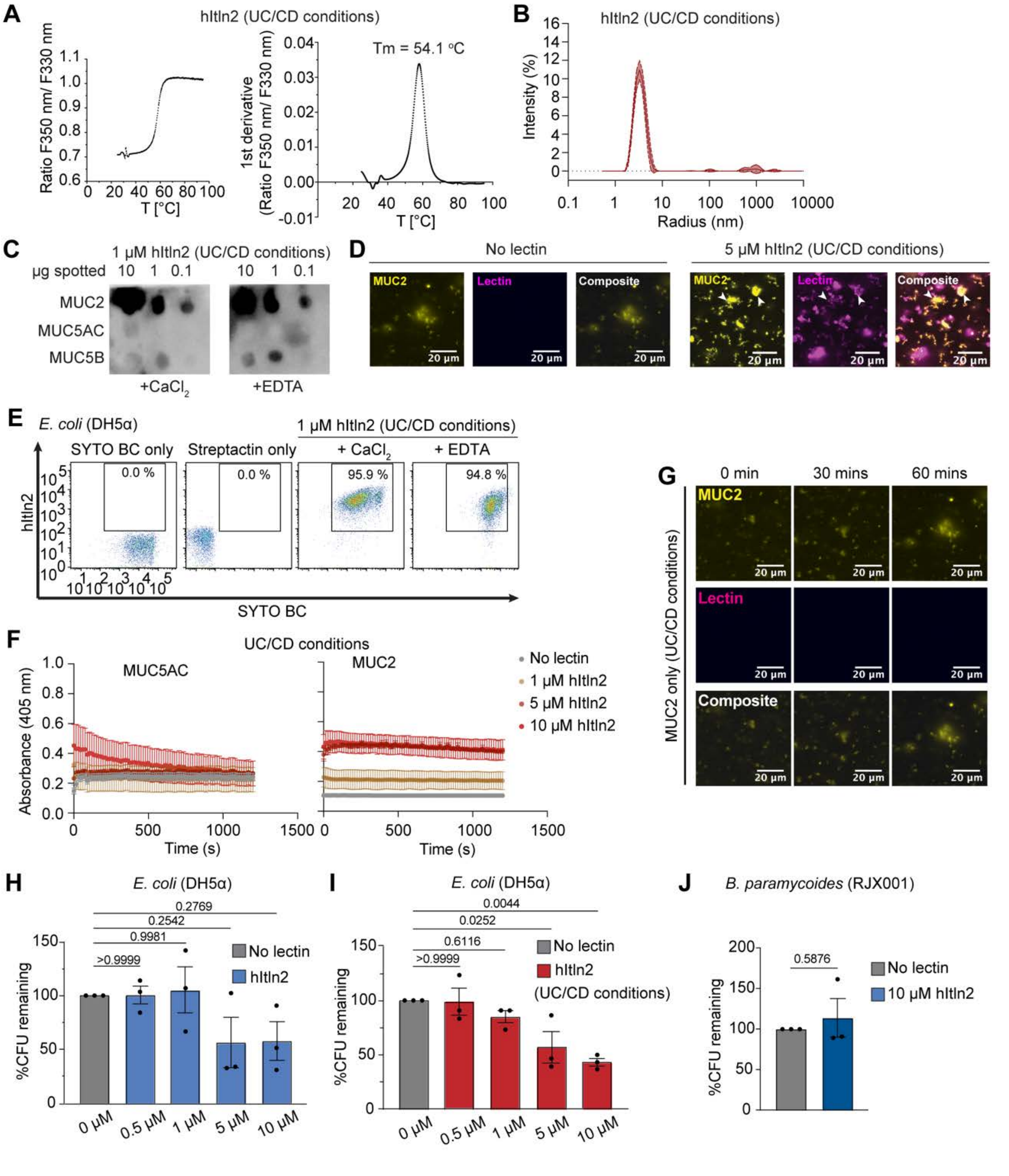
A
hItln2 (UC/CD conditions)
Ratio F350 nm/ F330 nm
T [°C]
1st derivative (Ratio F350 nm/ F330 nm)
Tm = 54.1 °C
B
hItln2 (UC/CD conditions)
Intensity (%)
Radius (nm)
C
1 μM hItln2 (UC/CD conditions)
μg spotted
MUC2
MUC5AC
MUC5B
+CaCl2
+EDTA
D
No lectin
5 μM hItln2 (UC/CD conditions)
MUC2
Lectin
Composite
E
E. coli (DH5α)
SYTO BC only
Streptactin only
1 μM hItln2 (UC/CD conditions)
+ CaCl2
+ EDTA
0.0 %
0.0 %
95.9 %
94.8 %
hItln2
SYTO BC
F
UC/CD conditions
MUC5AC
MUC2
Absorbance (405 nm)
Time (s)
No lectin
1 μM hItln2
5 μM hItln2
10 μM hItln2
G
0 min
30 mins
60 mins
MUC2 only (UC/CD conditions)
MUC2
Lectin
Composite
H
E. coli (DH5α)
%CFU remaining
No lectin
hItln2
0.2769
0.2542
0.9981
>0.9999
0 μM
0.5 μM
1 μM
5 μM
10 μM
I
E. coli (DH5α)
0.0044
0.0252
0.6116
>0.9999
No lectin
hItln2
(UC/CD conditions)
J
B. paramycoides (RJX001)
0.5876
No lectin
10 μM hItln2

Supplementary Fig. 11: Characterization of hItln2 activity at low pH and low salt (UC/CD conditions).

(**A**) Ratio of change in intrinsic fluorescence intensity at 330 nm and 350 nm (left) and its first derivative (right) as a function of temperature for recombinant hItln2 at low pH and salt concentration (UC/CD conditions), obtained from differential scanning fluorimetry. (**B**) Relative distribution of particle sizes for hItln2 protein at low pH and salt concentration (UC/CD conditions), analyzed by dynamic light scattering. (**C**) Dot blot analysis of MUC2, MUC5AC, and MUC5B probed with 1 µM hItln2 in Ca^{2+} or EDTA under low pH and salt concentration (UC/CD conditions), detected by Strep-HRP antibody. (**D**) Images of 1 µM hItln2 (magenta) binding to 0.01% (w/v) fluorescently labeled MUC2 (yellow) under low pH and salt concentration (UC/CD conditions), detected by Strep antibody. MUC2 without lectin treatment served as a control. Scale bars, 20 µm. **(E)** Flow cytometry of 1 µM hItln2 (UC/CD) binding to *E. coli* DH5α (E) in Ca^{2+} and EDTA conditions under low pH and salt concentrations (UC/CD conditions), detected by Streptactin. Unstained samples (no lectin) or those treated with SYTO BC, Strep antibody, or Streptactin alone served as controls. (**F**) Spectroscopic assay of 0.05% (w/v) MUC5AC and MUC2 crosslinking by hItln2, measured by the increase in absorbance at 405 nm. Data are shown as mean ± SD (n = 3 technical replicates). (**G**) Time-lapse images of 0.01% (w/v) fluorescently labeled MUC2 (yellow) without any mItln2 (magenta) treatment as a control for experiment depicted in Fig. 5G. Binding of hItln2 was detected with Strep antibody. Scale bars, 20 µm. (**H** and **I**) Quantification of viable *E. coli* DH5α by dilution plating after 4-hours incubation with various concentrations of hItln2 under physiological pH and salt conditions (H) and under low pH and salt concentration (UC/CD conditions) (I). Data show mean ± SEM (n = 3 independent experiments). One-way ANOVA followed by Dunnett's multiple comparisons test was used for (H) and (I). (**J**) Quantification of viable *B. paramycoides* RJX001 after 4-hours treatment with 10 µM hItln2. Data show mean ± SEM (n = 3 independent experiments; unpaired two-tailed t-test. Results in (A), (B), (C), and (E), are representative of three independent experiments. Result in (D), (F), and (G) are representative of two independent experiments. Source data are found in the source data file.

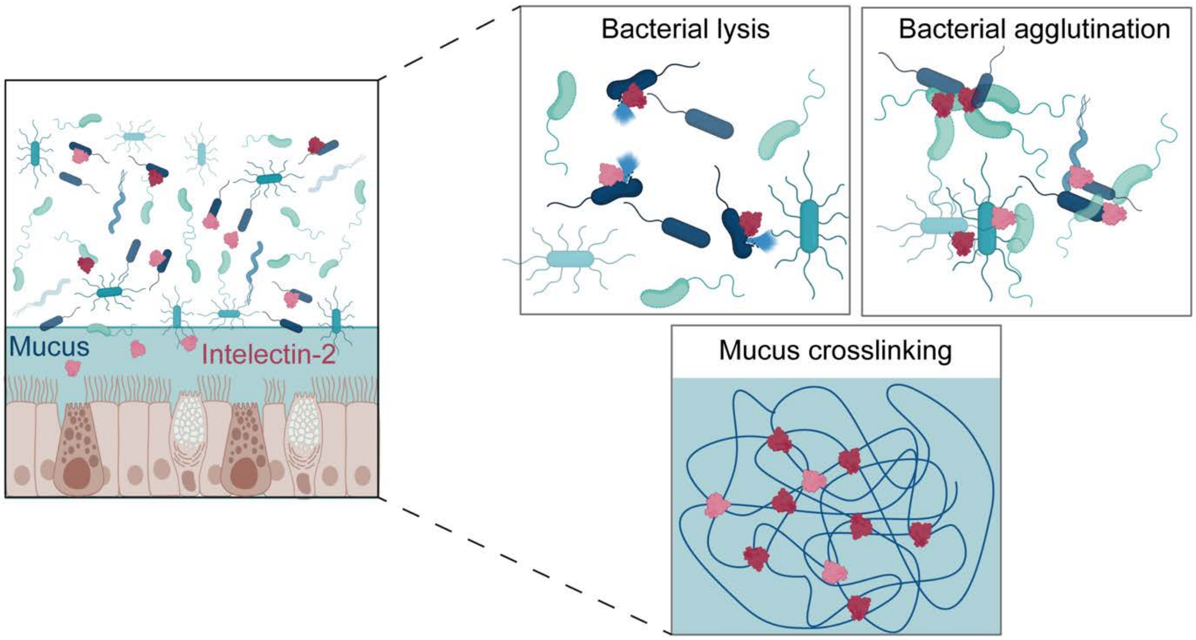

Supplementary Fig. 12: Proposed role of intelectin-2 in mucosal homeostasis.
Created in BioRender. Lab01, K. (2025) https://BioRender.com/umppvti.

**Read Count (% total)**	**SI (tissue)**	**CTRL (enteroids)**	**IL-4 (enteroids)**	**IL-13 (enteroids)**	**IL-22 (enteroids)**
Total	815974	1509287	1590206	1180500	1238489
Itln1	773631 (94.8%)	1464695 (97.0%)	1102941 (69.4%)	810944 (68.7%)	1203291 (97.2%)
Itln2	42343 (5.2%)	44592 (3.0%)	487265 (30.6%)	369556 (31.3%)	35198 (2.8%)
Itln3	ND	ND	ND	ND	ND
Itln4	ND	ND	ND	ND	ND
Itln5	ND	ND	ND	ND	ND
Itln6	ND	ND	ND	ND	ND

Supplementary Table 1. Read count (mRNA) and relative abundance of intelectin paralogs from small intestine (SI) and enteroids derived from B6.C-Itln1-6 mice, as determined by next-generation sequencing. CTRL = control, ND = not detected.

Separate Files

Supplementary Data 1. Mouse intelectin-2 binding specificity as determined from the microbial (from CFG) and mammalian (from CFG and RayBiotech) glycan microarrays.

Supplementary Data 2. Human intelectin-2 binding specificity as determined from the microbial glycan microarray (CFG).

Legends for microbial (from CFG) and mammalian (from RayBiotech) glycan arrays

Supplementary Movie 1. Timelapse of *L. reuteri* (untreated). Live *L. reuteri* (brightfield) stained with SYTO BC (green) was treated with HEPES/Ca/BSA/T buffer containing StrepMAB Classic-649 (red) and imaged every 10 minutes over 6 hours. Scale bars = 20 µm.

Supplementary Movie 2. Timelapse of *L. reuteri* (mItln2 treated). Live *L. reuteri* (brightfield) stained with SYTO BC (green) was treated with HEPES/Ca/BSA/T buffer containing 5 µM StrepII-mItln2 labeled with StrepMAB Classic-649 (red) and imaged every 10 minutes over 6 hours. Scale bars = 20 µm.

Supplementary Movie 3. Timelapse of *L. reuteri* (mItln2 treated). Live *L. reuteri* (brightfield) stained with SYTO BC (green) was treated with HEPES/Ca/BSA/T buffer containing 5 µM StrepII-mItln2 labeled with StrepMAB Classic-649 (red) and imaged every 10 minutes over 6 hours to visualize decrease in SYTO BC fluorescence after mItln2 treatment. Scale bars = 50 µm.

Source Data for Supplemental Figures:

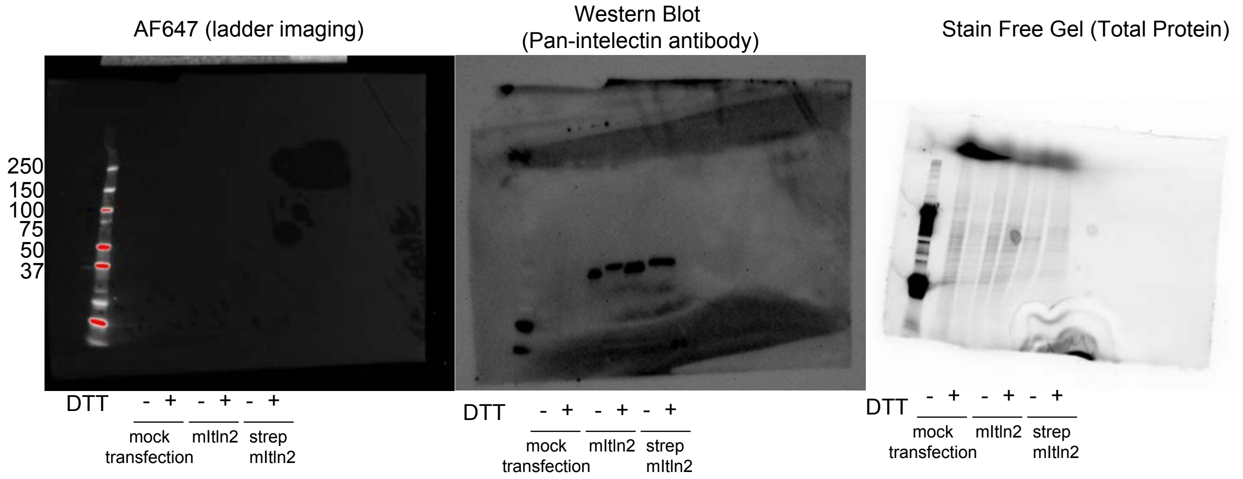

Source Data for Figure S1D. Western blot (middle) and corresponding stain-free gel (far-right) of the culture medium from HEK293T cells transfected with native mItln2 (no-tag) or StrepII-tagged mItln2 run under reducing and non-reducing conditions. Recombinant mItln2 was detected using a pan-intelectin antibody.

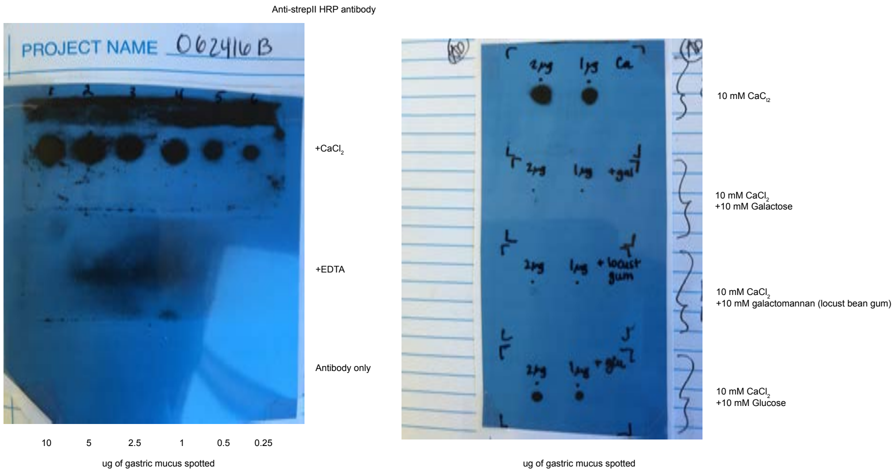

Source Data for Figure S3L and S3M. Dot blot analysis for different amounts of gastric mucus on nitrocellulose membrane was performed with 0.5 µM mItln2, either in the presence of Ca^{2+} or EDTA. Controls included no lectin and treatment with 10 mM carbohydrates (galactose or glucose). Binding of mItln2 was detected using a Strep-HRP antibody.

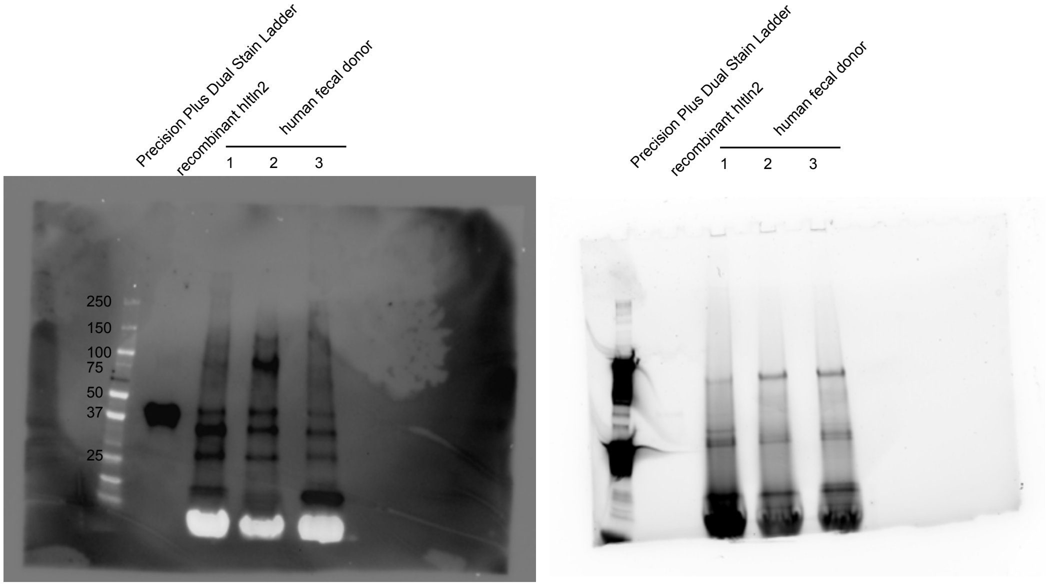

Composite of anti-Itln2 Western Blot (Bevins Antibody) and Coomassie (for ladder detection)

Stain-Free Gel Imaging (total protein)

Source Data for Figure S8B. Western blot (left) and corresponding stain-free gel (right) of fecal samples from three individuals under reducing conditions. In western blot, hItln2 was detected by an intelectin-2 specific antibody. Recombinant StrepII-tagged hItln2 was used as a control.

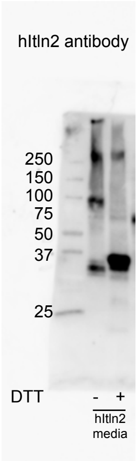

Source Data for Figure S8F. Western blot of hItln2 protein under reducing or non-reducing conditions, visualized with anti-intelectin2 antibody. Molecular weight of monomeric hItln2 is 34 kDa.

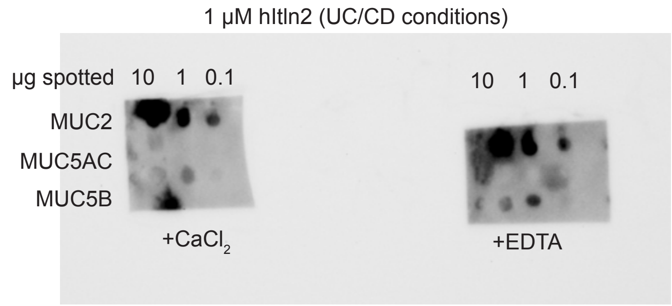

Source Data for Figure S11C. Dot blot analysis of MUC2, MUC5AC, and MUC5B probed with 1 μM hItln2 in Ca^{2+} or EDTA under low pH and salt concentration (UC/CD conditions), detected by Strep-HRP antibody.
