# Supplementary material for: Intelectin-2 is a broad-spectrum antimicrobial lectin
Source: Nat Commun. 2026 Jan 13;17:231. doi: 10.1038/s41467-025-67099-4 (PMC12800186; doi:10.1038/s41467-025-67099-4)
Supplement: Supplementary file 2 — Description of Additional Supplementary Files [file 41467_2025_67099_MOESM2_ESM.pdf]

Title: Supplementary Data 1

Description: Mouse intelectin-2 binding specificity as determined from the microbial (from CFG) and mammalian (from CFG and RayBiotech) glycan microarrays.

Title: Supplementary Data 2

Description: Human intelectin-2 binding specificity as determined from the microbial glycan microarray (CFG). Legends for microbial (from CFG) and mammalian (from RayBiotech) glycan arrays

Title: Supplementary Movie 1

Description: Timelapse of L. reuteri (untreated). Live L. reuteri (brightfield) stained with SYTO BC (green) was treated with HEPES/Ca/BSA/T buffer containing StrepMAB Classic-649 (red) and imaged every 10 minutes over 6 hours. Scale bars = 20 μm.

Title: Supplementary Movie 2

Description: Timelapse of L. reuteri (mItln2 treated). Live L. reuteri (brightfield) stained with SYTO BC (green) was treated with HEPES/Ca/BSA/T buffer containing 5 μM StrepII-mItln2 labeled with StrepMAB Classic-649 (red) and imaged every 10 minutes over 6 hours. Scale bars = 20 μm.

Title: Supplementary Movie 3

Description: Timelapse of L. reuteri (mItln2 treated). Live L. reuteri (brightfield) stained with SYTO BC (green) was treated with HEPES/Ca/BSA/T buffer containing 5 μM StrepII-mItln2 labeled with StrepMAB Classic-649 (red) and imaged every 10 minutes over 6 hours to visualize decrease in SYTO BC fluorescence after mItln2 treatment. Scale bars = 50 μm.
