# Supplementary material for: Intelectin-2 is a broad-spectrum antimicrobial lectin
Source: Nat Commun. 2026 Jan 13;17:231. doi: 10.1038/s41467-025-67099-4 (PMC12800186; doi:10.1038/s41467-025-67099-4)

Legends for glycans displayed in CFG microbial microarray

Chart#	BPS#	BACTERIA / STRAIN	NAME / STRUCTURE /Cat.No.	STRUCTURE
1	1	Providencia stuartii O49	PO49 Core-linked	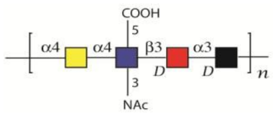
2	2	Providencia stuartii O52	PO52 Core-linked	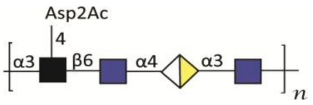
3	3	Pseudomonas aerguinosa O4 (Habs serotype 4)	PO4 Core-linked	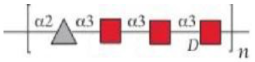
4	4	Pseudomonas aerguinosa O1 (Fisher immunotype 4)	PO1 Core-linked	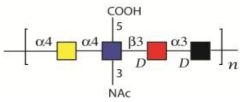
5	5	Pseudomonas aerguinosa O2 (Fisher immunotype 3)	PO2 Core-linked	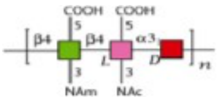
6	6	Pseudomonas aerguinosa O13 (Sandvik serotype II)	PO13 Core-linked	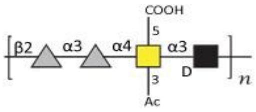
7	7	Pseudomonas aerguinosa O9 (9a, 9b, 9d)	PO9 Core-linked	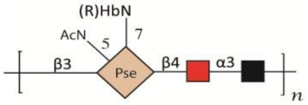
8	8	Pseudomonas aerguinosa O6a (Habs serotype6, fraction IIa)	PO6a Core-linked- O-unit	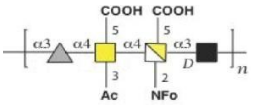
9	9	Pseudomonas aerguinosa O6a (Habs serotype6, fraction IIb)	PO6a unsubstituted core	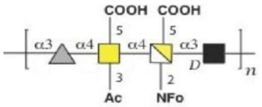

10	12	Salmonella typhimurium SL 11881 (Re mut)	LPS-L9516	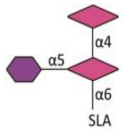
11	13	Salmonella typhimurium TV 119 (Ra mut)	LPS-L6016	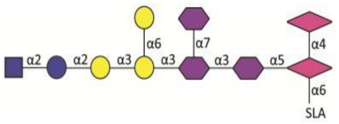
12	14	Salmonella typhimurium SL 684 (Rc mut)	LPS-L5891	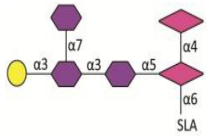
13	15	Pseudomonas aerguinosa O10	L8643	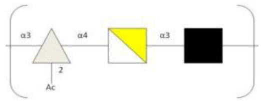
14	16	Salmonella typhimurium dodeca saccharide	4809	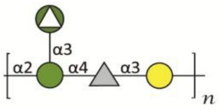
15	17	Salmonella enteritidis dodeca saccharide	1262	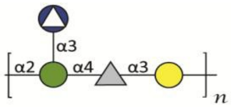
16	18	Salmonella typhimurium LPS	L2262	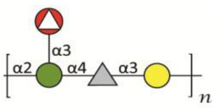
17	20	Serratia marcescens LPS	L6136	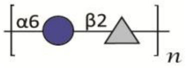
18	22	Escherichia coli K235 LPS	L2143	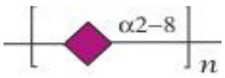
19	23	Escherichia coli O128-B12 LPS	L2755	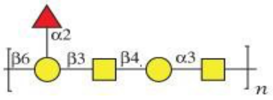
20	25	Salmonella enterica abortus equi LPS	L5886	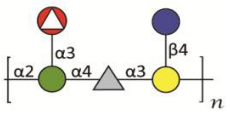

21	26	Salmonella typhosa LPS	L2387	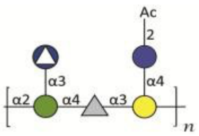
22	27	Salmonella enteritidis LPS	L2012	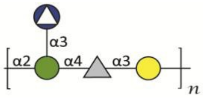
23	28	Shigella bodyii type2		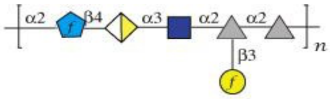
24	29	Shigella bodyii type4		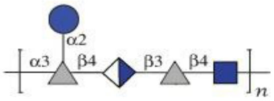
25	30	Shigella bodyii type10		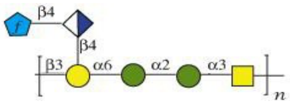
26	31	Shigella dysennteriae type 3		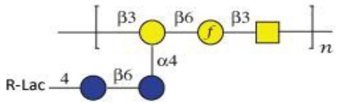
27	32	Shigella dysennteriae type 8 (batch 12)		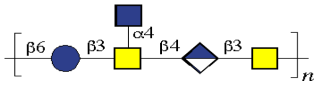
28	33	Shigella dysennteriae type 11		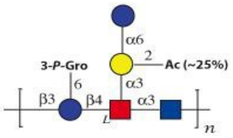
29	34	Shigella dysennteriae type 13		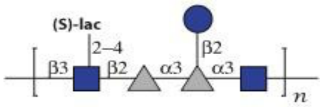
30	35	Escherichia coli O29		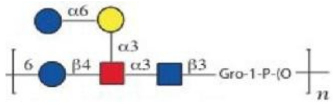

31	36	Escherichia coli O40		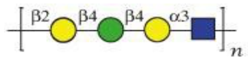
32	37	Escherichia coli O106		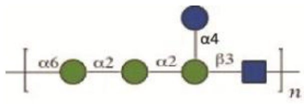
33	38	Escherichia coli O130		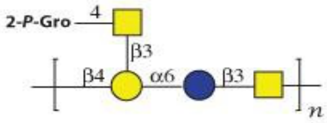
34	39	Escherichia coli O148		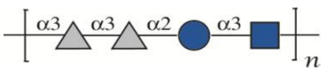
35	40	Escherichia coli O150		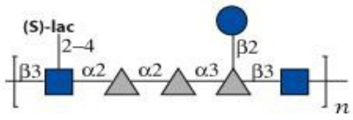
36	41	Escherichia coli O180		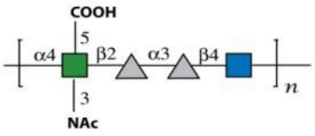
37	42	Proteus mirabilis O3a, 3c (G1)		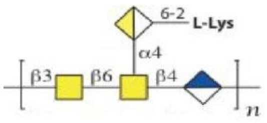
38	43	Proteus mirabilis O8 (TG326)		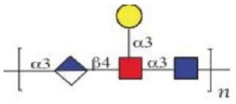
39	44	Proteus mirabilis O10 (HJ4320)		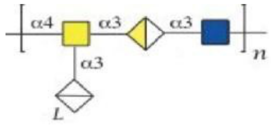
40	45	Proteus mirabilis O29a, 29b (2002)		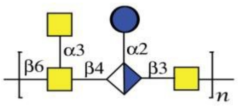
41	46	Proteus mirabilis O50 (TG332)		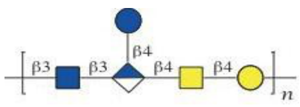

42	47	Proteus mirabilis O54a, 54b (10704)		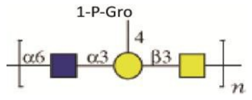
43	48	Proteus mirabilis O57 (TG319)		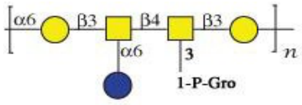
44	49	Proteus penneri O8 (106)		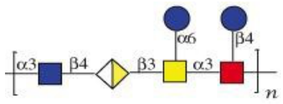
45	50	Proteus penneri O64a, 64b, 64d (39)		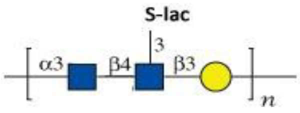
46	51	Proteus penneri O66 (2)		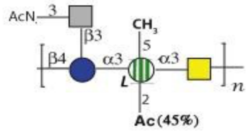
47	52	Proteus penneri O69 (25)		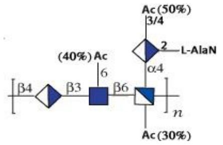
48	53	Proteus penneri O71 (42)		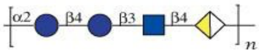
49	54	Proteus penneri O72a, 72b (4)		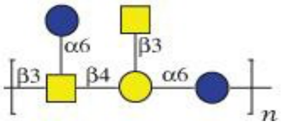
50	55	Pseudomonas aerguinosa O2 (2a),2d,2f	IATS 10 , OPS	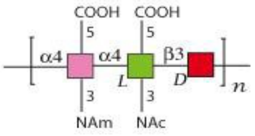
51	56	Pseudomonas aerguinosa O2 2a,2b	IATS 16 OPS	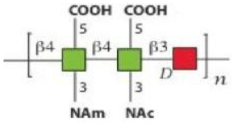
52	57	Pseudomonas aerguinosa O2 2a,2b,2e	IATS NO, OPS	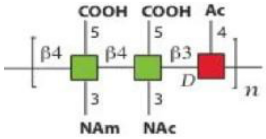

53	58	Pseudomonas aerguinosa O2 2a,2d	IATS 5 OPS	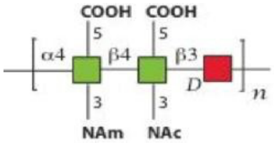
54	59	Pseudomonas aerguinosa O2 Immuno 7	IATS 18, OPS	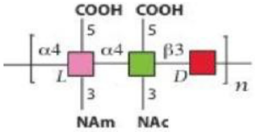
55	60	Pseudomonas aerguinosa O3 3a,3b	IATS NO, OPS	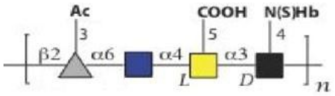
56	61	Pseudomonas aerguinosa O3 3a,3b,3c	IATS 3, OPS	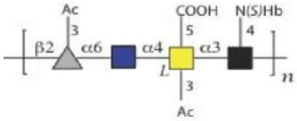
57	62	Pseudomonas aerguinosa O3 3a,3d	IATS NO, OPS	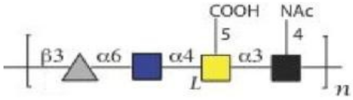
58	63	Pseudomonas aerguinosa O4 4a,4c	IATS NO, OPS	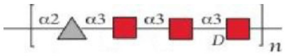
59	64	Pseudomonas aerguinosa O6 6a	IATS 6, OPS	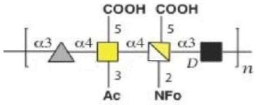
60	65	Pseudomonas aerguinosa O6 6a,6c	IATS NO, OPS	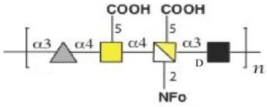
61	66	Pseudomonas aerguinosa O6 Immuno 1	IATS NO, OPS	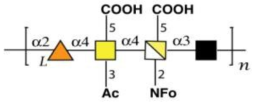
62	67	Pseudomonas aerguinosa O7 7a,7b,7c	IATS 7,LPS	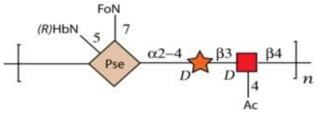
63	68	Pseudomonas aerguinosa O7 7a,7b,7d	IATS 8,LPS	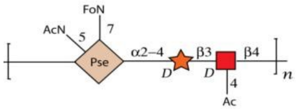

64	69	Pseudomonas aerguinosa O7 7a,7d	IATS NO, LPS	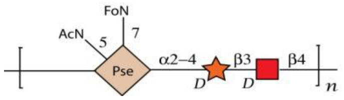
65	71	Pseudomonas aerguinosa O10 10a,10b	IATS 10, OPS	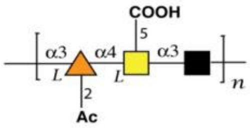
66	72	Pseudomonas aerguinosa O10 10a,10c	IATS 19, OPS	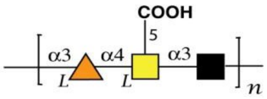
67	73	Pseudomonas aerguinosa O11 11a,11b	IATS 11, OPS	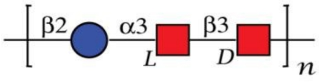
68	74	Pseudomonas aerguinosa O12 12	IATS 12, OPS<br>Habs 12	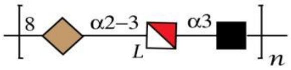
69	75	Pseudomonas aerguinosa O13 13a,13c	IATS 14, OPS	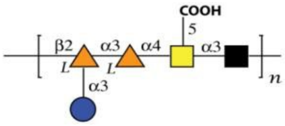
70	76	Pseudomonas aerguinosa O14 14	IATS 17,OPS<br>Meitert X	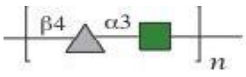
71	77	Pseudomonas aerguinosa O15 15	IATS 15, OPS	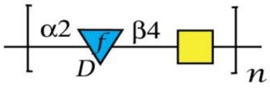
72	78	Proteus vulgaris O1 (18984)*	LPS	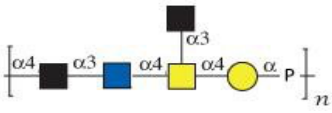
73	79	Proteus vulgaris O4 (PrK 9/57)	OPS	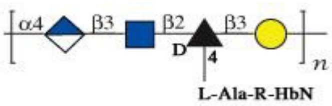
74	80	Proteus vulgaris O12 (PrK 25/57)	OPS	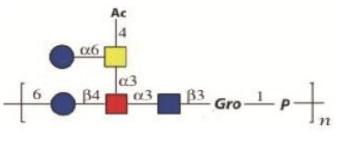

75	81	Proteus vulgaris O13 (8344)	OPS	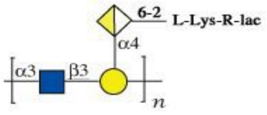
76	82	Proteus vulgaris O15 (PrK 30/57)	OPS	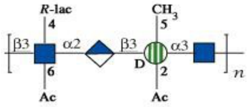
77	83	Proteus vulgaris O17 (PrK 33/57)	OPS	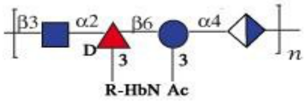
78	84	Proteus vulgaris O19a (PrK 37/57)	OPS	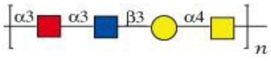
79	85	Proteus vulgaris O21 (PrK 39/57)*	LPS	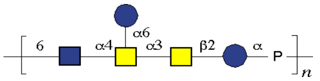
80	86	Proteus vulgaris O22 (PrK 40/57)	OPS	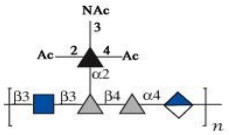
81	88	Proteus vulgaris O25 (PrK 48/57)	OPS	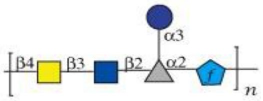
82	89	Proteus vulgaris O34 (4669)*	LPS	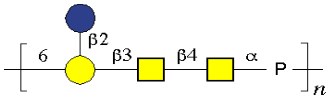
83	90	Proteus vulgaris O37a,b (PrK 63/57)	OPS	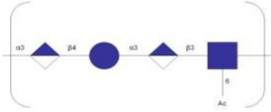
84	91	Proteus vulgaris O37a,c (PrK 72/57)	OPS	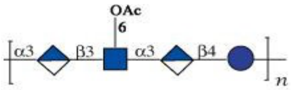
85	92	Proteus vulgaris O44 (PrK 67/57)	OPS	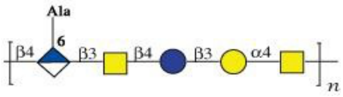

86	93	Proteus vulgaris O45 (4680)	OPS	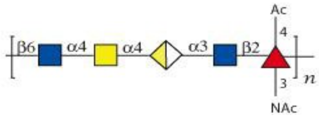
87	94	Proteus vulgaris O53 (TG 276- 10)	OPS	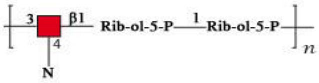
88	95	Proteus vulgaris O54a,54c (TG 103)	OPS	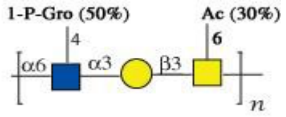
89	96	Proteus vulgaris O55 (TG 155)	OPS	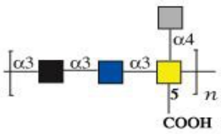
90	97	Proteus vulgaris O65 (TG 251)	OPS	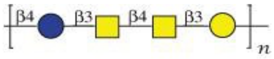
91	98	Proteus mirabilis O6 (PrK 14/57)	OPS	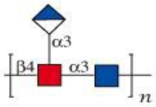
92	99	Proteus mirabilis O11 (PrK 24/57)	OPS	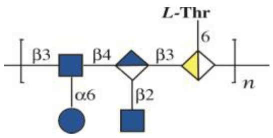
93	100	Proteus mirabilis O13 (PrK 26/57)	OPS	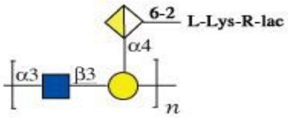
94	101	Proteus mirabilis O14a,14b (PrK 29/57)	OPS	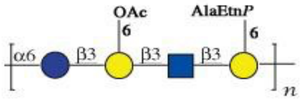
95	102	Proteus mirabilis O16 (4652)	OPS	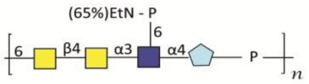
96	103	Proteus mirabilis O17 (PrK 32/57)	OPS	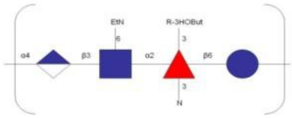

97	104	Proteus mirabilis O23a,b,d (PrK 42/57)	OPS	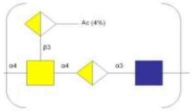
98	106	Proteus mirabilis O26 (PrK 49/57)	OPS	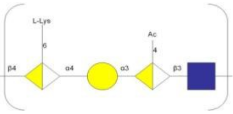
99	107	Proteus mirabilis O27 (PrK 50/57)	OPS	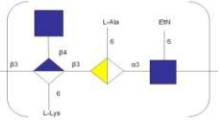
100	108	Proteus mirabilis O28 (PrK 51/57)	OPS	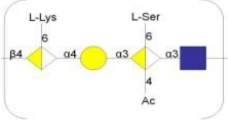
101	109	Proteus mirabilis O29a (PrK 52/57)	OPS	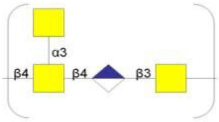
102	110	Proteus mirabilis O40 (10703)	OPS	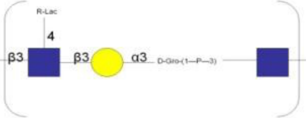
103	111	Proteus mirabilis O41 (PrK 67/57)	OPS	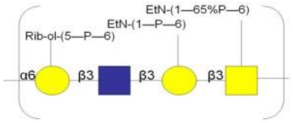
104	112	Proteus mirabilis O51 (19011)*	LPS	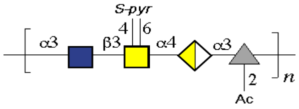
105	113	Proteus mirabilis O74 (10705, OF)	OPS	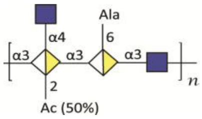
106	114	Proteus mirabilis O75 (10702, OC)	OPS	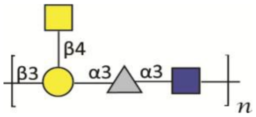
107	115	Proteus mirabilis O77 (3 B-m)	OPS	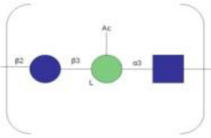

108	116	Proteus penneri O31a (26)	OPS	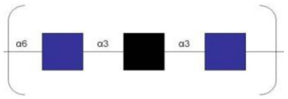
109	117	Proteus penneri O52 (15)	OPS	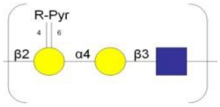
110	118	Proteus penneri O58 (12)	OPS	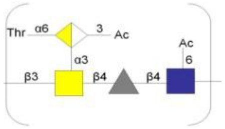
111	119	Proteus penneri O59 (9)	OPS	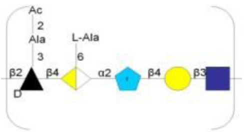
112	120	Proteus penneri O61 (21)	OPS	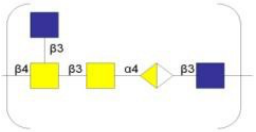
113	121	Proteus penneri O62 (41)	OPS	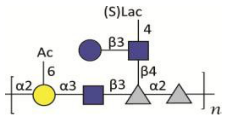
114	122	Proteus penneri O63 (22)	OPS	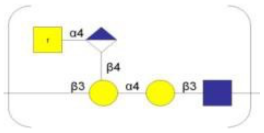
115	123	Proteus penneri O64a,b,c (27)	OPS	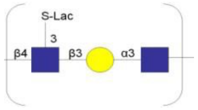
116	124	Proteus penneri O65 (34)	OPS	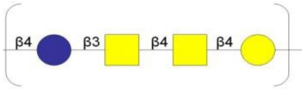
117	125	Proteus penneri O67 (8)	OPS	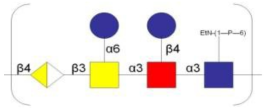
118	126	Proteus penneri O68 (63)	OPS	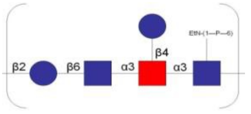

119	127	Proteus penneri O70 (60)	OPS	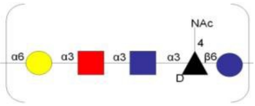
120	128	Proteus penneri O73a,b (103)	OPS	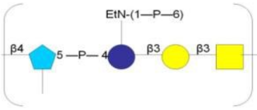
121	129	Proteus myxofaciens O60	OPS	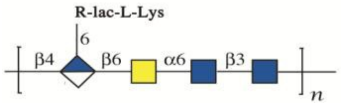
122	130	Proteus O56 (genomospecies 4)	OPS	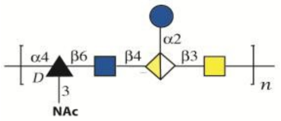
123	131	Providencia stuartii O4	OPS	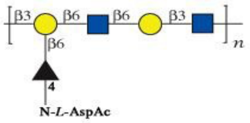
124	132	Providencia stuartii O18	OPS	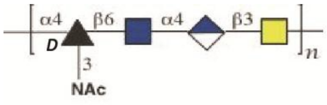
125	133	Providencia stuartii O20*	LPS	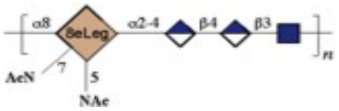
126	134	Providencia stuartii O43	OPS	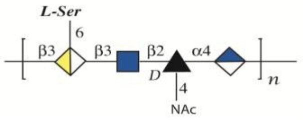
127	135	Providencia stuartii O44	OPS	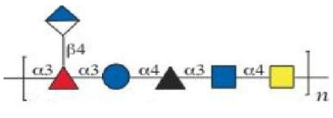
128	136	Providencia stuartii O47	OPS	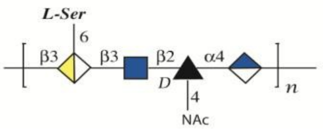
129	137	Providencia stuartii O47, Core 9	OPS	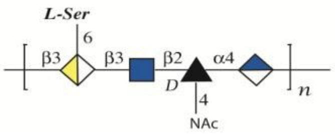

130	138	Providencia stuartii O49, Core 1	OPS	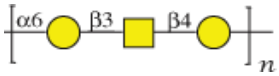
131	139	Providencia stuartii O57	OPS	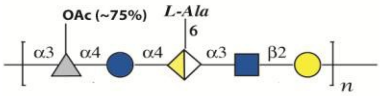
132	140	Providencia alcalifaciens O5	OPS	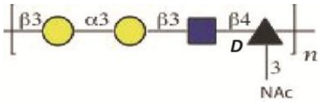
133	141	Providencia alcalifaciens O6*	LPS	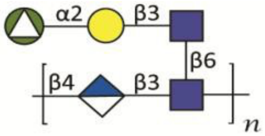
134	142	Providencia alcalifaciens O19	OPS	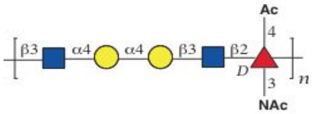
135	143	Providencia alcalifaciens O19	LPS	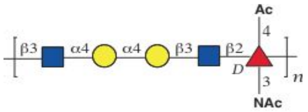
136	144	Providencia alcalifaciens O19	LPS/NaOH	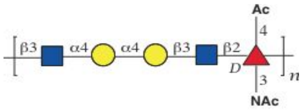
137	145	Providencia alcalifaciens O21	OPS	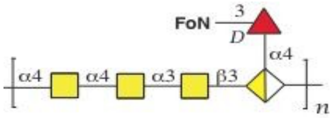
138	146	Providencia alcalifaciens O23	OPS	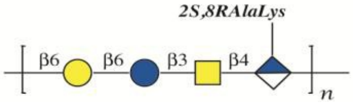
139	147	Providencia alcalifaciens O27	OPS	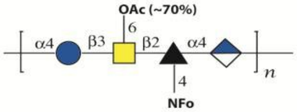
140	148	Providencia alcalifaciens O29	OPS	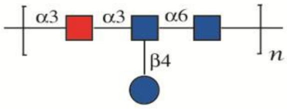

141	149	Providencia alcalifaciens O30	OPS	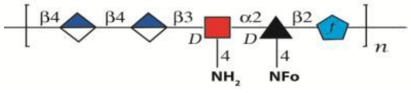
142	150	Providencia alcalifaciens O32	OPS	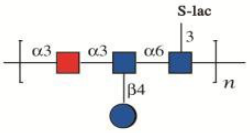
143	151	Providencia alcalifaciens O36*	LPS-NH4OH	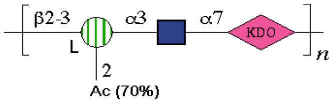
144	152	Providencia alcalifaciens O39	OPS	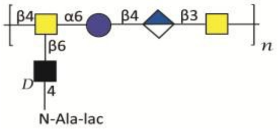
145	153	Providencia rustigianii O14	OPS	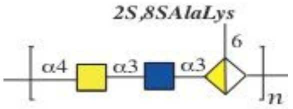
146	154	Providencia rustigianii O16	OPS	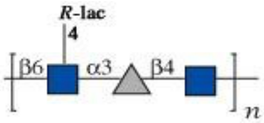
147	155	Providencia rustigianii O34	OPS	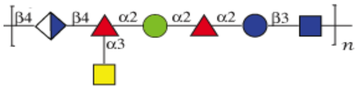
148	156	Yersinia pestis, KM260(11)-∆0187	LPS	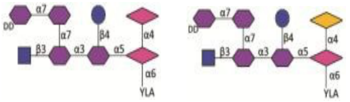
149	157	Yersinia pestis, KM260(11)-∆0187	Core oligo saccharide	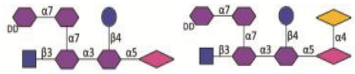
150	158	Yersinia pestis, KM260(11)-∆rfe	LPS	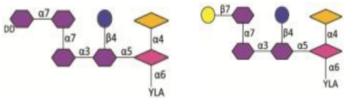
151	159	Yersinia pestis, KM260(11)-∆rfe	Core oligo saccharide	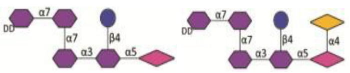

152	160	Yersinia pestis, 1146-25	LPS	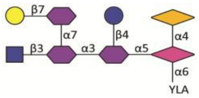
153	161	Yersinia pestis 1146-25	Core oligo saccharide	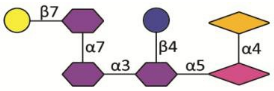
154	162	Yersinia pestis, 1146-37	LPS	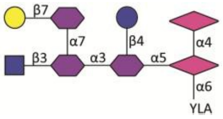
155	163	Yersinia pestis, 1146-37	Core oligo saccharide	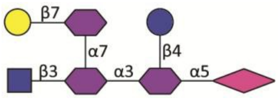
156	164	Yersinia pestis, KM218-37	LPS	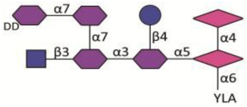
157	165	Yersinia pestis, KM218-37	Core oligo saccharide	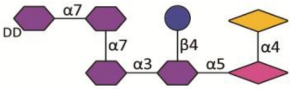
158	166	Yersinia pestis, KM218-25	LPS	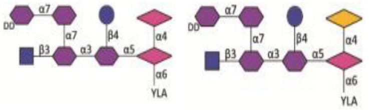
159	167	Yersinia pestis, KM218-25	Core oligo saccharide	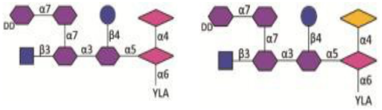
160	168	Yersinia pestis, KM260(11)-∆pmrF	LPS	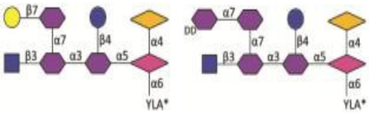
161	169	Yersinia pestis, KM260(11)-∆pmrF	Core oligo saccharide	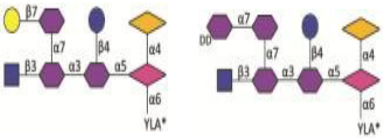
162	170	Yersinia pestis, KM260(11)-∆0186	LPS	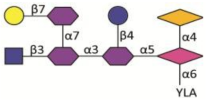

163	171	Yersinia pestis, KM260(11)-∆0186	Core oligo saccharide	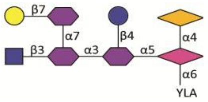
164	172	Yersinia pestis, KM260(11)-∆waaQ	LPS	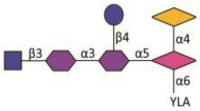
165	173	Yersinia pestis, KM260(11)-∆waaQ	Core oligo saccharide	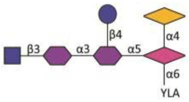
166	174	Yersinia pestis, KM260(11)-∆waaL	LPS	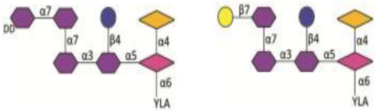
167	175	Yersinia pestis, KM260(11)-25	LPS	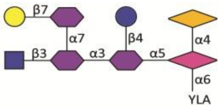
168	176	Yersinia pestis, KM260(11)-25	Core oligo saccharide	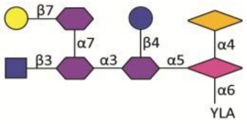
169	177	Yersinia pestis, KM260(11)-37	Core oligo saccharide	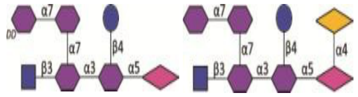
170	178	Yersinia pestis, KIMD1-37	Core oligo saccharide	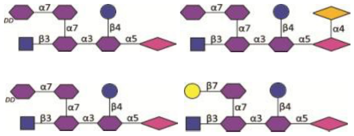
171	179	Yersinia pestis, KIMD1-25	Core oligo saccharide	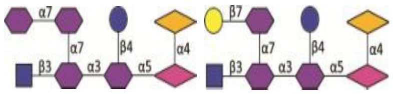
172	180	Yersinia pestis, 11M-25	LPS	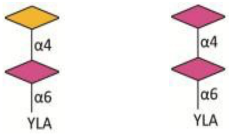
173	181	Yersinia pestis, 11M-37	LPS	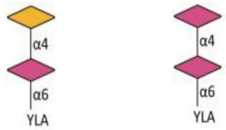

174	182	Proteus vulgaris O23a, 23b, 23c (CCUG 10701)	OPS	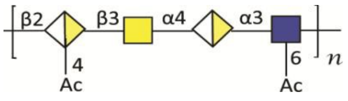
175	183	Proteus vulgaris O24 (PrK 47/57)	LPSOH	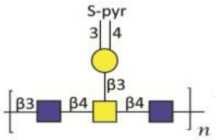
176	184	Yersinia pestis KM260(11)-6C	LPS	
177	185	Yersinia pestis 260(11)-37C-186	LPS	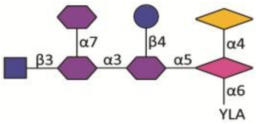
178	186	Yersinia pestis 260(11)-37C-187	LPS	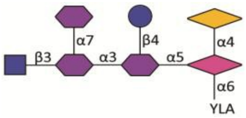
179	187	Yersinia pestis 260(11)-37C-416	LPS	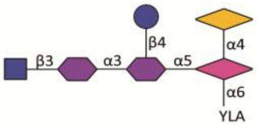
180	188	Yersinia pestis 260(11)-37C-417	LPS	
181	189	Yersinia pestis P-1680-25C	OS	
182	190	Yersinia pestis P-1680-37C	LPS	
183	191	Yersinia pestis I-2377-25C	OS	
184	192	Yersinia pestis I-2377-37C	LPS	

185	193	Francisella novicida OPS	OPS	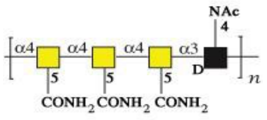
186	194	Francisella tularensis OPS	OPS	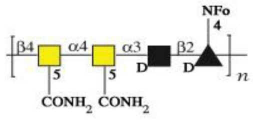
187	195	Klebsiella O1 OPS	OPS	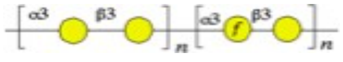
188	196	Klebsiella O2a OPS	OPS	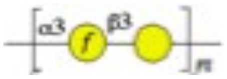
189	197	Klebsiella O2ac OPS	OPS	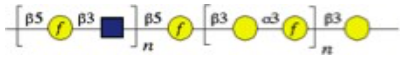
190	198	Klebsiella O3 OPS	OPS	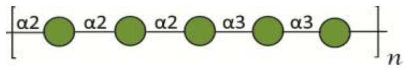
191	199	Klebsiella O4 OPS	OPS	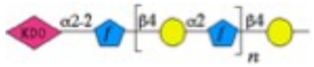
192	200	Klebsiella O5 OPS	OPS	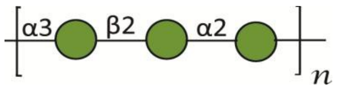
193	201	Klebsiella O8 OPS	OPS	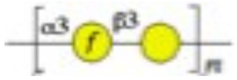
194	202	Klebsiella O12 OPS	OPS	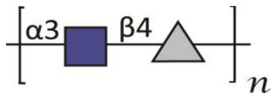
195	203	Shigella boydii type 1	LPSOH	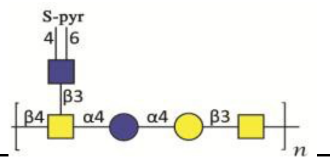

196	204	Shigella boydii type 3	OPS	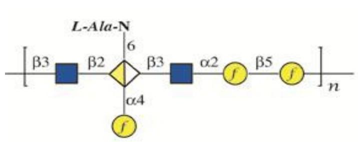
197	205	Shigella boydii type 5	OPS	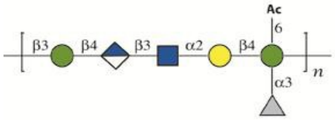
198	206	Shigella boydii type 9	OPS	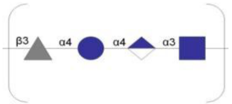
199	207	Shigella boydii type 11	OPS	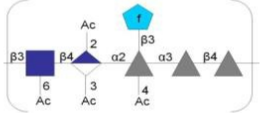
200	208	Shigella boydii type 12	OPS	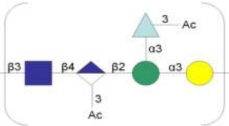
201	209	Shigella boydii type 15	OPS	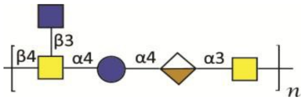
202	210	Shigella boydii type 16	OPS	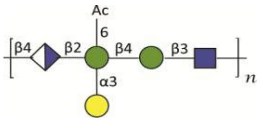
203	211	Shigella boydii type 17	OPS	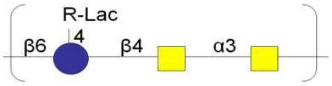
204	212	Shigella boydii type 18	OPS	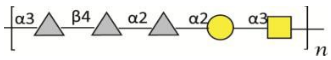
205	213	Escherichia coli O49	OPS	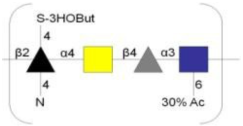
206	214	Escherichia coli O52	OPS	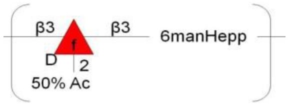

207	215	Escherichia coli O58	OPS	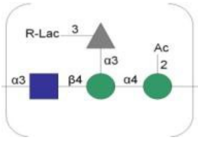
208	216	Escherichia coli O61	LPSOH	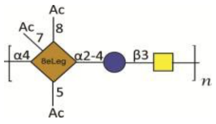
209	217	Escherichia coli O73	OPS	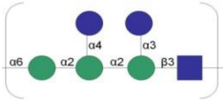
210	218	Escherichia coli O112ab	OPS	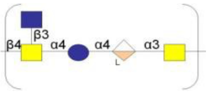
211	219	Escherichia coli O118	OPS	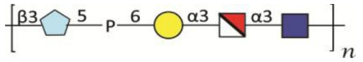
212	220	Escherichia coli O125	OPS	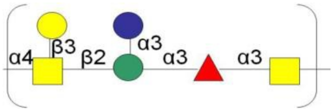
213	221	Escherichia coli O151	OPS	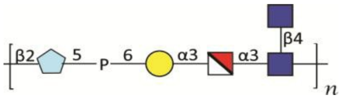
214	222	Escherichia coli O168	OPS	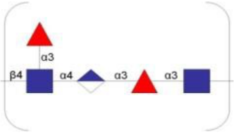
215	223	Shigella dysenteriae type 2	LPSOH	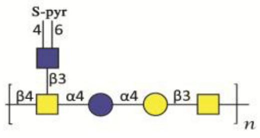
216	224	Shigella dysenteriae type 4	OPS	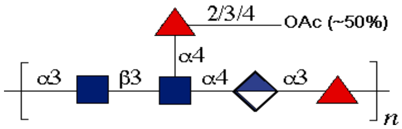
217	225	Shigella dysenteriae type 5	OPS	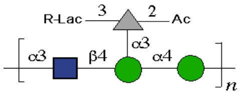

218	226	Shigella dysenteriae type 6 SR- strain	SR-strain	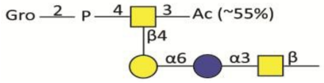
219	227	Shigella dysenteriae type 7	OPS	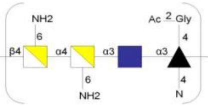
220	228	Shigella dysenteriae type 8 (Russian)	OPS	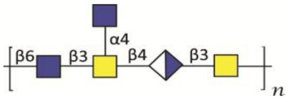
221	229	Shigella dysenteriae type 9	OPS	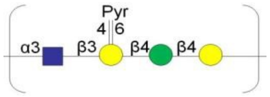
222	231	Escherichia coli O111:B4 LPS-solution at 1 mg/mL	L5293-2ML (LPS) (Sigma)	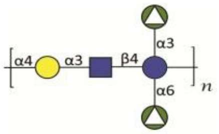
223	232	Escherichia coli O26:B6 LPS-solution at 1 mg/mL	L5543-2ML (LPS) (Sigma)	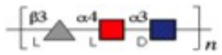
224	233	Escherichia coli O55:B5 LPS-solution at 1 mg/mL	L5418-2ML (LPS) (Sigma)	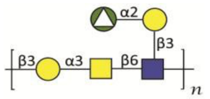
225	234	Escherichia coli O127:B8 LPS-solution at 1 mg/mL	L5668-2ML (LPS) (Sigma)	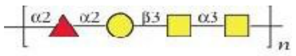
226	235	Streptococcus pneumoniae type 1 (Danish type 1)	161-X // Capsular PS	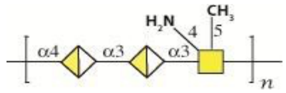
227	236	Streptococcus pneumoniae type 2 (Danish type 2)	165-X// Capsular PS	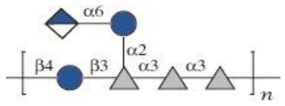
228	237	Streptococcus pneumoniae type 3 (Danish type 3)	169-X// Capsular PS	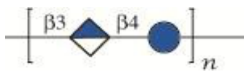

229	238	Streptococcus pneumoniae type 4 (Danish type 4)	173-X// Capsular PS	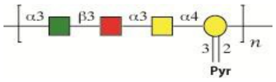
230	239	Streptococcus pneumoniae type 5 (Danish type 5)	177-X// Capsular PS	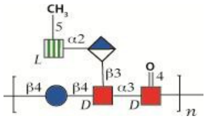
231	240	Streptococcus pneumoniae type 8 (Danish type 8)	185-X// Capsular PS	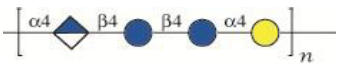
232	241	Streptococcus pneumoniae type 9 (Danish type 9N)	189-X// Capsular PS	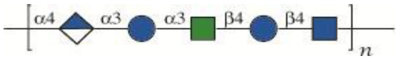
233	242	Streptococcus pneumoniae type 12 (Danish type 12F)	193-X// Capsular PS	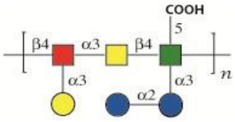
234	243	Streptococcus pneumoniae type 14 (Danish type 14)	197-X// Capsular PS	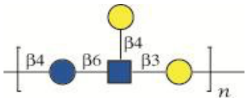
235	244	Streptococcus pneumoniae type 17 (Danish type 17F)	201-X// Capsular PS	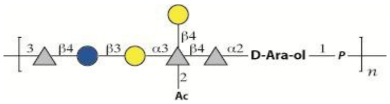
236	245	Streptococcus pneumoniae type 19 (Danish type 19F)	205-X// Capsular PS	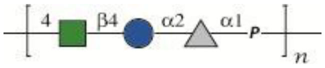
237	246	Streptococcus pneumoniae type 20 (Danish type 20)	209-X// Capsular PS	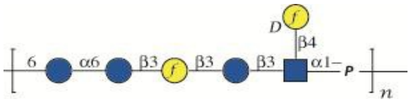
238	247	Streptococcus pneumoniae type 22 (Danish type 22F)	213-X// Capsular PS	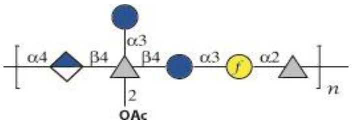
239	248	Streptococcus pneumoniae type 23 (Danish type 23F)	217-X// Capsular PS	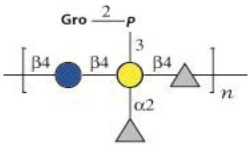

240	249	Streptococcus pneumoniae type 26 (Danish type 6B)	225-X// Capsular PS	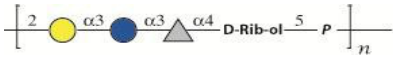
241	250	Streptococcus pneumoniae type 34 (Danish type 10A)	229-X// Capsular PS	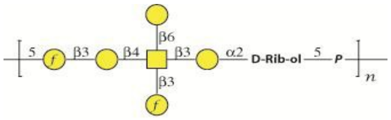
242	251	Streptococcus pneumoniae type 43 (Danish type 11A)	233-X// Capsular PS	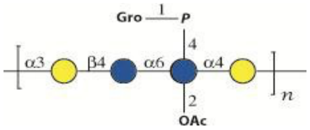
243	252	Streptococcus pneumoniae type 51 (Danish type 7F)	237-X// Capsular PS	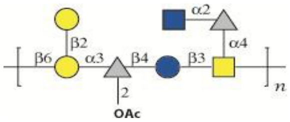
244	253	Streptococcus pneumoniae type 54 (Danish type 15B)	241-X// Capsular PS	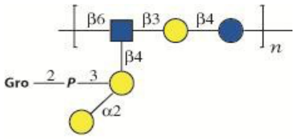
245	254	Streptococcus pneumoniae type 56 (Danish type 18C)	245-X// Capsular PS	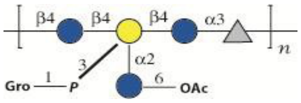
246	255	Streptococcus pneumoniae type 57 (Danish type 19A)	249-X// Capsular PS	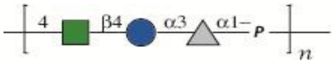
247	256	Streptococcus pneumoniae type 68 (Danish type 9V)	253-X// Capsular PS	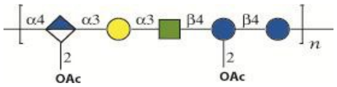
248	257	Streptococcus pneumoniae type 70 (Danish type 33F)	257-X// Capsular PS	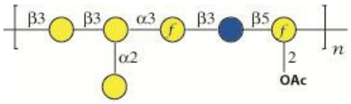
249	258	Yersinia pestis KM218-6C	OS	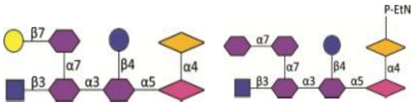
250	259	Yersinia pestis KM260(11)-yjhW- 6C	OS	

251	260	Yersinia pestis KM260(11)-wabD/waaL	OS	
252	261	Yersinia pestis KM260(11)-wabC/waaL	OS	
253	262	Yersinia pseudotuberculosis 85pCad-37C	OS	
254	263	Yersinia pseudotuberculosis 85pCad-20C	OS	
255	264	Yersinia pseudotuberculosis O:2a	PS	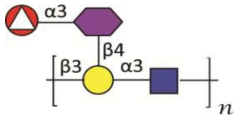
256	265	Yersinia pseudotuberculosis O:2a-dhmA	PS	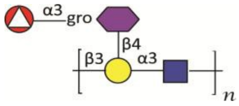
257	266	Yersinia pseudotuberculosis O:2c	PS	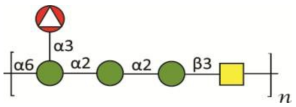
258	267	Yersinia pseudotuberculosis O:3	PS	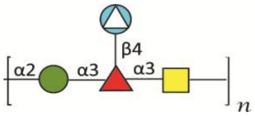
259	268	Yersinia pseudotuberculosis O:4b	PS	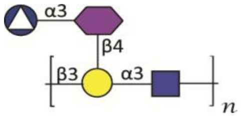
260	269	Proteus vulgaris O2 (OX2)	PS	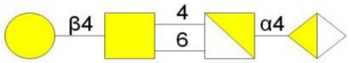
261	270	Proteus mirabilis O3ab (S1959)	PS	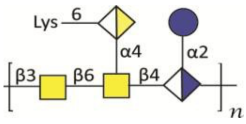

262	271	Proteus mirabilis O5 (PrK 12/57)	PS	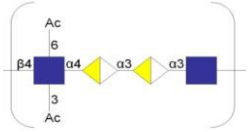
263	272	Proteus mirabilis O9 (PrK 18/57)	PS	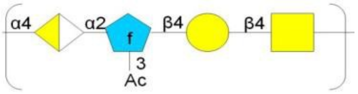
264	273	Proteus mirabilis O11 (9B-m)	PS	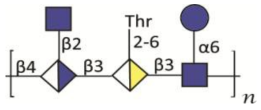
265	274	Proteus penneri O17 (16)	PS	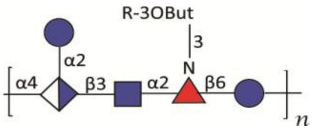
266	275	Proteus mirabilis O18 (PrK 34/57)	LPSOH	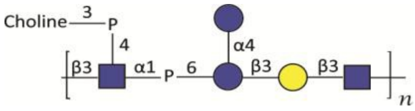
267	276	Proteus mirabilis O20 (PrK 38/57)	LPSOH	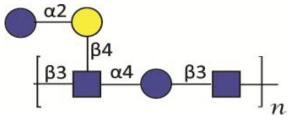
268	277	Proteus penneri O31ab (28)	PS	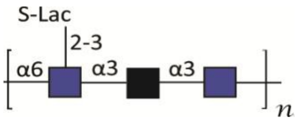
269	278	Proteus mirabilis O33 (D52)	PS	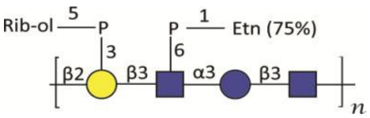
270	279	Proteus mirabilis O43 (PrK 69/57)	PS	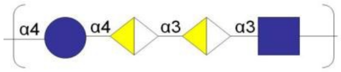
271	280	Proteus vulgaris O47 (PrK 73/57)	Not stated	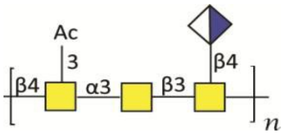
272	281	Proteus mirabilis O49 (PrK 75/57)	PS	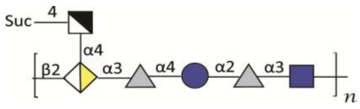

273	282	Proteus mirabilis O54ab (OE)	PS	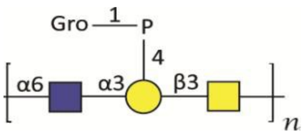
274	283	Proteus penneri O73ac (75)	PS	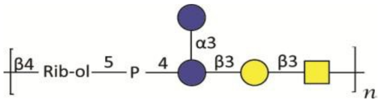
275	284	Proteus vulgaris O76 (HSC438)	PS	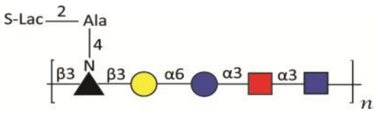
276	285	Shigella flexneri type 1a	PS	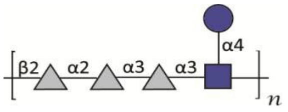
277	286	Shigella flexneri type 1b	PS	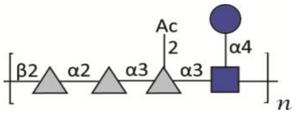
278	287	Shigella flexneri type 2a	PS	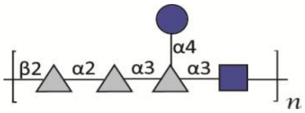
279	288	Shigella flexneri type 2b	PS	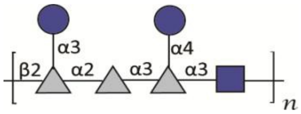
280	289	Shigella flexneri type 3a	PS	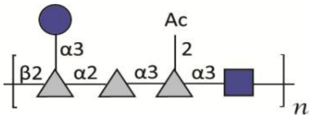
281	290	Shigella flexneri type 3b	PS	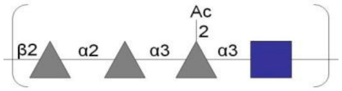
282	291	Shigella flexneri type 4a	PS	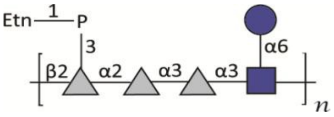
283	292	Shigella flexneri type 4b	PS	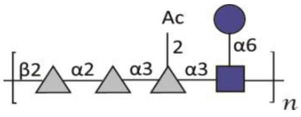

284	293	Shigella flexneri type 5b	PS	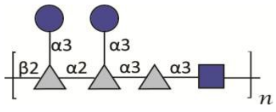
285	294	Shigella flexneri type 6a	PS	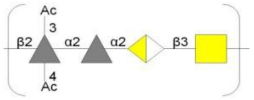
286	295	Shigella flexneri type 6	PS	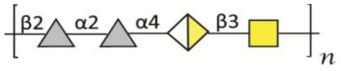
287	296	Shigella flexneri type X	PS	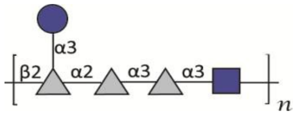
288	297	Shigella dysenteriae type 1	PS	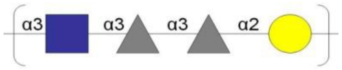
289	298	Shigella boydii type 6	PS	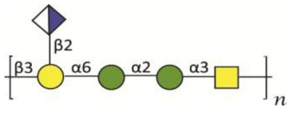
290	299	Shigella boydii type 7	PS	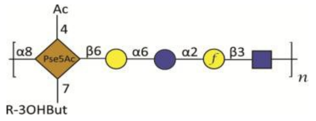
291	300	Shigella boydii type 8	PS	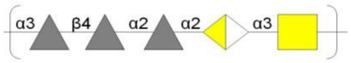
292	301	Shigella boydii type 13	LPSOH	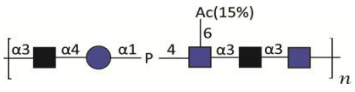
293	302	Shigella boydii type 14	LPSOH	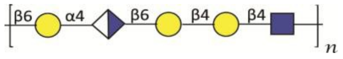
294	303	Escherichia coli O71	PS	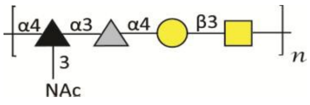

295	304	Escherichia coli O85	PS	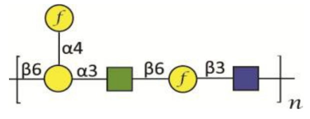
296	305	Escherichia coli O99	PS	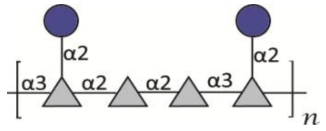
297	306	Escherichia coli O145	LPSOH	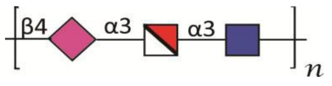
298	307	Escherichia coli O107	PS	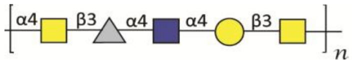
299	308	Salmonella enterica O17	PS	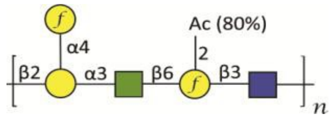
300	309	Salmonella enterica O28	PS	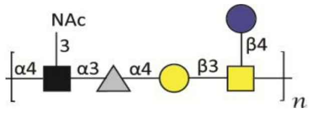
301	310	Salmonella enterica O47	PS	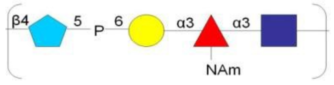
302	311	Salmonella enterica O55	PS	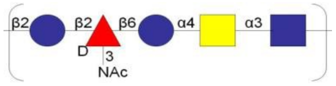
303	312	Escherichia coli K92	CPS	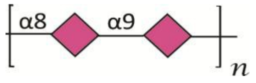
304	313	Escherichia coli K5	CPS	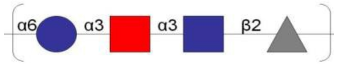
305	314	Escherichia coli K13	CPS	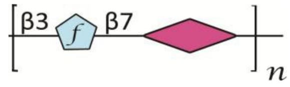

306	315	Neisseria meningitidis Group C	CPS	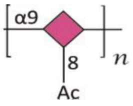
307	316	Davanat		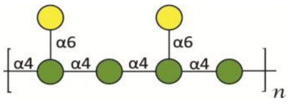
308	317	Laminarin		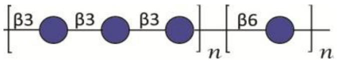
309	318	Yeast Mannan		
310	319	Escherichia coli O86		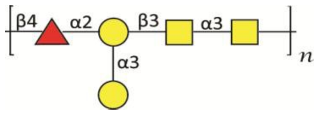
311	320	Galactomannan DAVANT (160102) Pro-Pharmacenti		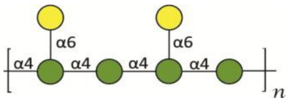
312	321	Yeast Mannan Sigma M-3640		
313	322	1-2 Mannan Acetobacter methanolicus MB135		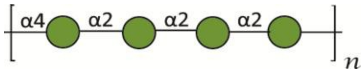

Legends for glycans displayed in RayBiotech microarray

Glycan 300 Structure Symbols

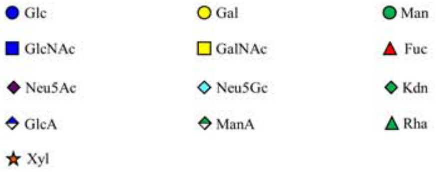

Glycan 100 Glycans

A. Linkers

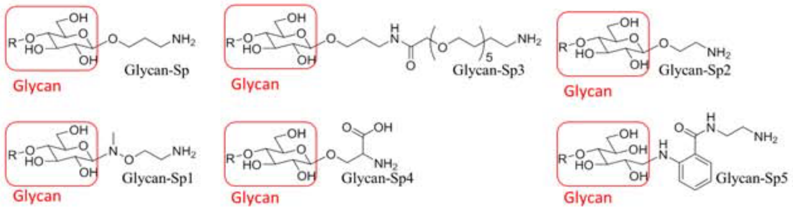

B. Glycan 100 Structures

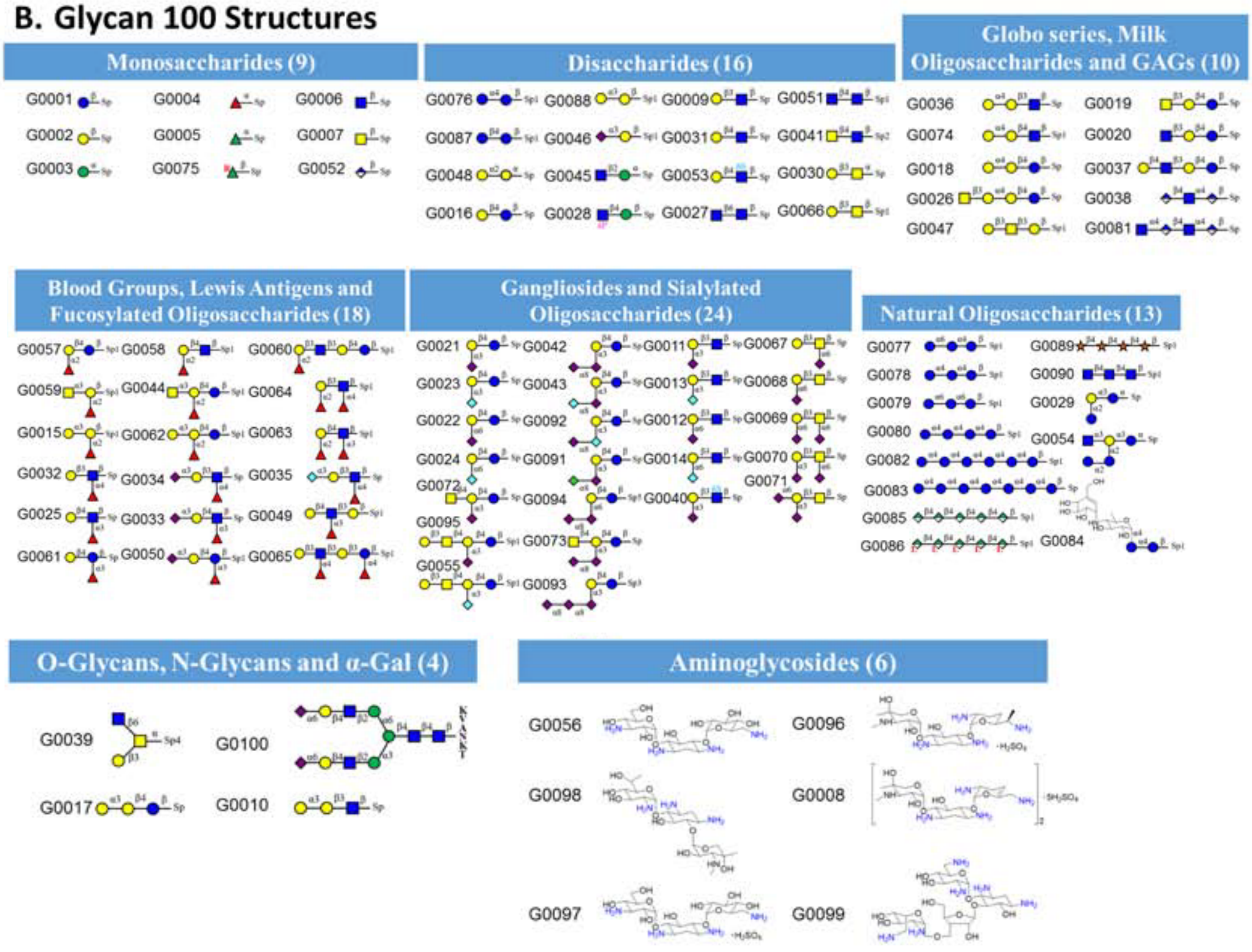

N-Glycans

A. Linkers

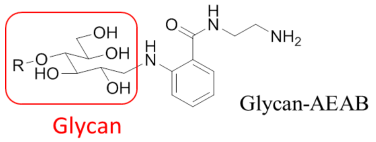

B. N-glycan structures

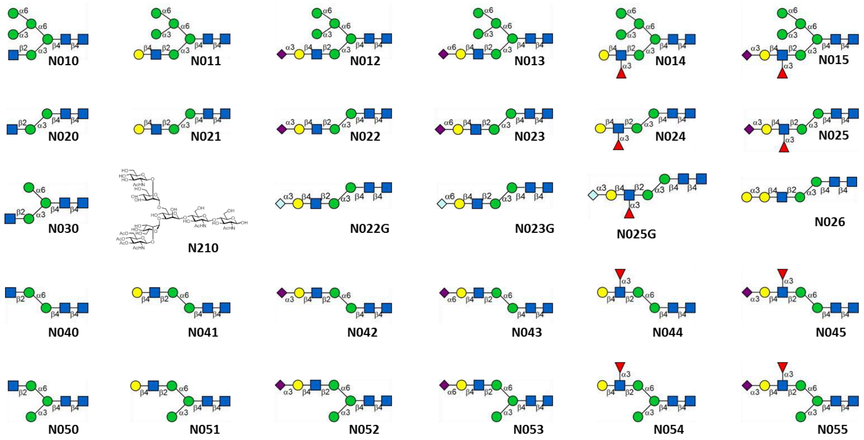

Glycolipid Glycans

A. Linkers

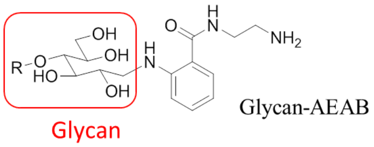

B. Glycolipid Glycan Structures

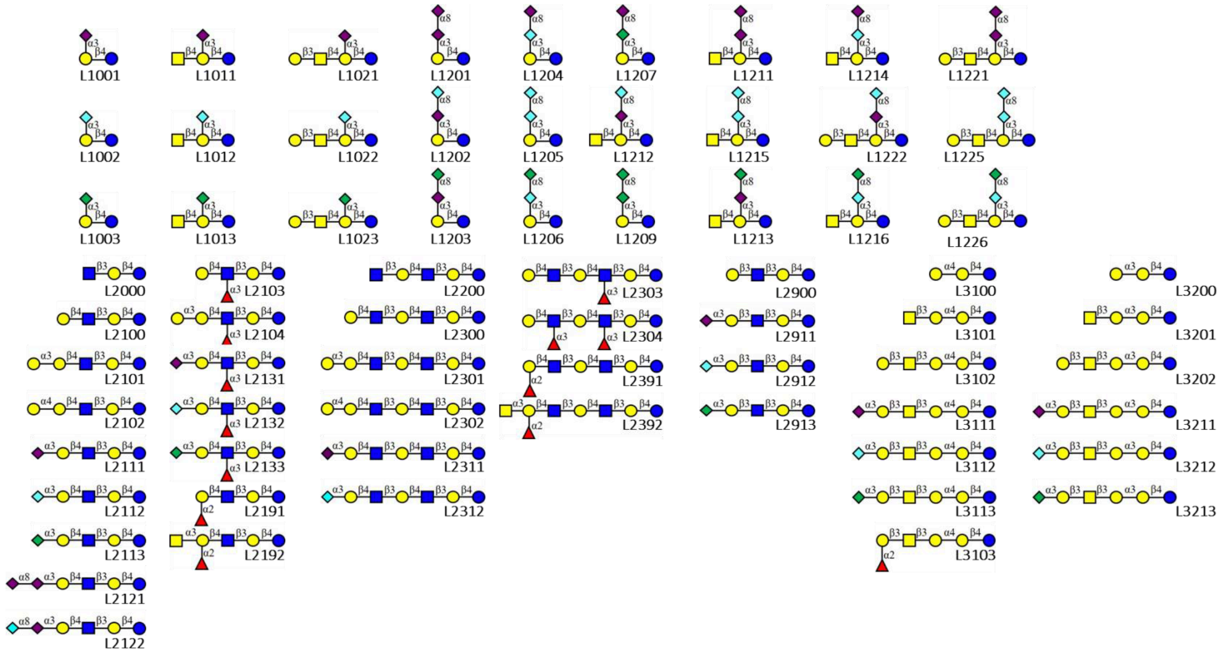

Human Milk Oligosaccharides

A. Linkers

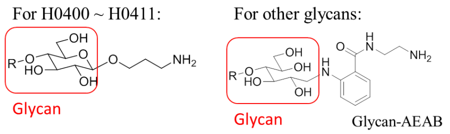

B. Human Milk Oligosaccharide Structures

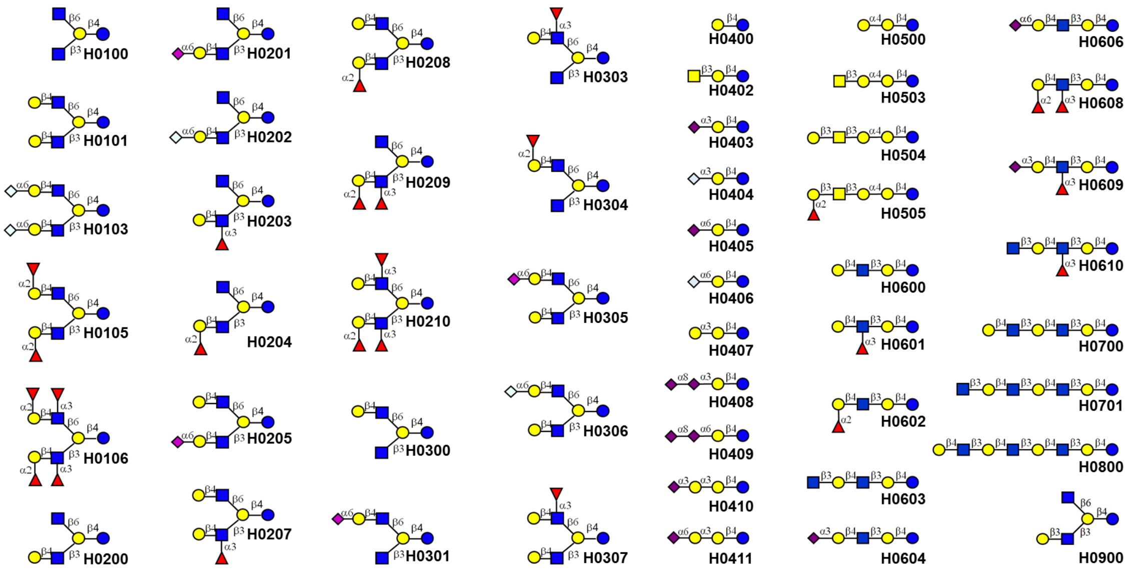

Tandem Epitopes

A. Linkers

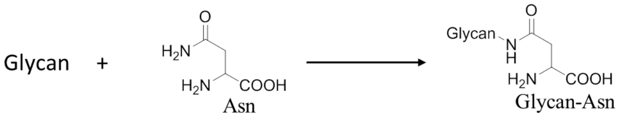

B. Tandem Epitope Structures

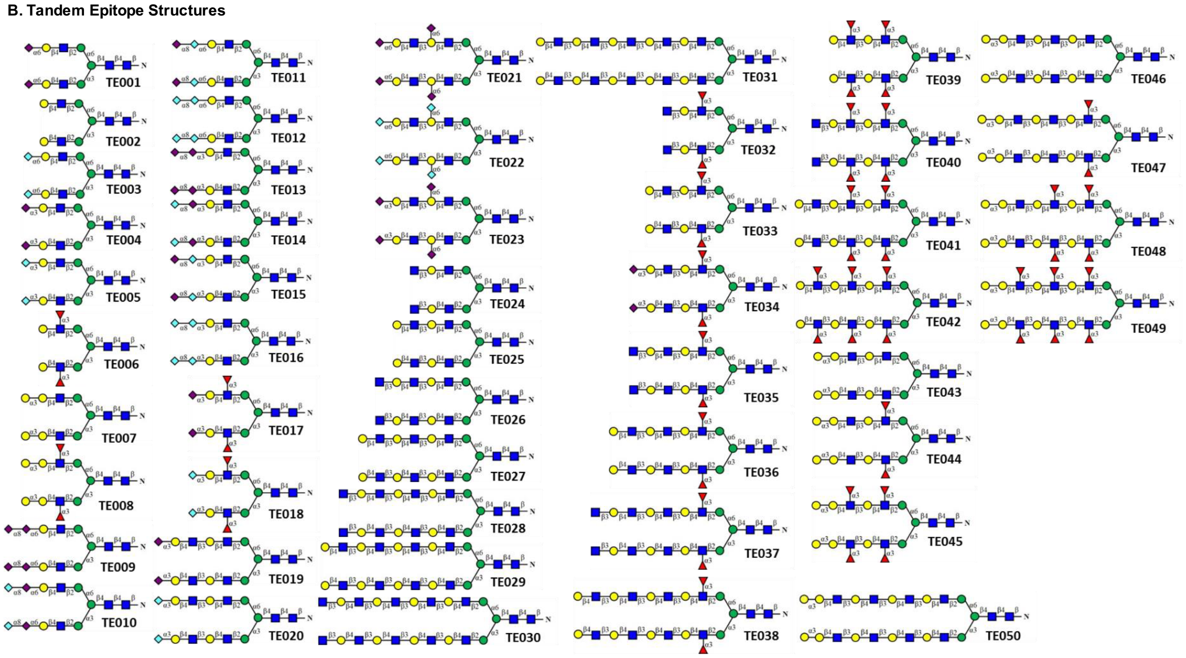

Supplement: Supplementary file 5 — Supplementary Dataset 3 [file 41467_2025_67099_MOESM5_ESM.pdf]
