# Supplementary material for: Intelectin-2 is a broad-spectrum antimicrobial lectin
Source: Nat Commun. 2026 Jan 13;17:231. doi: 10.1038/s41467-025-67099-4 (PMC12800186; doi:10.1038/s41467-025-67099-4)
Supplement: Supplementary file 9 — Reporting Summary [file 41467_2025_67099_MOESM9_ESM.pdf]

nature portfolio

Corresponding author(s): Laura L. Kiessling

Last updated by author(s): 04/22/2025

Reporting Summary

Nature Portfolio wishes to improve the reproducibility of the work that we publish. This form provides structure for consistency and transparency in reporting. For further information on Nature Portfolio policies, see our Editorial Policies and the Editorial Policy Checklist.

Statistics

For all statistical analyses, confirm that the following items are present in the figure legend, table legend, main text, or Methods section.

n/a	Confirmed	
☐	☒	The exact sample size (*n*) for each experimental group/condition, given as a discrete number and unit of measurement
☐	☒	A statement on whether measurements were taken from distinct samples or whether the same sample was measured repeatedly
☐	☒	The statistical test(s) used AND whether they are one- or two-sided<br>*Only common tests should be described solely by name; describe more complex techniques in the Methods section.*
☒	☐	A description of all covariates tested
☐	☒	A description of any assumptions or corrections, such as tests of normality and adjustment for multiple comparisons
☐	☒	A full description of the statistical parameters including central tendency (e.g. means) or other basic estimates (e.g. regression coefficient) AND variation (e.g. standard deviation) or associated estimates of uncertainty (e.g. confidence intervals)
☐	☒	For null hypothesis testing, the test statistic (e.g. *F*, *t*, *r*) with confidence intervals, effect sizes, degrees of freedom and *P* value noted<br>*Give P values as exact values whenever suitable.*
☒	☐	For Bayesian analysis, information on the choice of priors and Markov chain Monte Carlo settings
☒	☐	For hierarchical and complex designs, identification of the appropriate level for tests and full reporting of outcomes
☒	☐	Estimates of effect sizes (e.g. Cohen's *d*, Pearson's *r*), indicating how they were calculated

Our web collection on statistics for biologists contains articles on many of the points above.

Software and code

Policy information about availability of computer code

Data collection	Molecular Devices IXM HC confocal microscope and Olympus FV1200 Laser Scanning Confocal Microscope were used for microscopy. ThermoFisher Attune NxT, BD Fortessa and BD Symphony were used for flow cytometry.
Data analysis	Protein structures and overlays were generated with PyMol (The PyMol Molecular Graphics System, Version 3.0 Schrodinger, LLC). Homology models of mItln2 and hItln2 were generated with AlphaFold3. STAT6 binding motif in the promoter sequences of mItln2 and hItln2 was analyzed with FIMO version 5.5.8. Flow cytometry data was analyzed using FlowJo (FlowJo™ v10.8 for Mac Software). Imaging data was analyzed using Fiji. GraphPad Prism version 10.1.1 for Mac was used for data analysis and graph generation (GraphPad Software, Boston, MA USA, www.graphpad.com). Sequencing data was analyzed using SnapGene (www.snapgene.com).

For manuscripts utilizing custom algorithms or software that are central to the research but not yet described in published literature, software must be made available to editors and reviewers. We strongly encourage code deposition in a community repository (e.g. GitHub). See the Nature Portfolio guidelines for submitting code & software for further information.

Data

Policy information about availability of data

All manuscripts must include a data availability statement. This statement should provide the following information, where applicable:

- Accession codes, unique identifiers, or web links for publicly available datasets
- A description of any restrictions on data availability
- For clinical datasets or third party data, please ensure that the statement adheres to our policy

The glycan array data for mItln2 and hItln2 (Fig. 2B, 5E, and S3B) are provided in the Supplementary Data 1 and Data 2. The glycan array data for hItln1 was obtained from doi: 10.1038/nsmb.3053. All data supporting the findings of this study are available within the article and the source data file. Any additional information required to reanalyze the data reported in this paper is available from the lead contact upon request.

Research involving human participants, their data, or biological material

Policy information about studies with human participants or human data. See also policy information about sex, gender (identity/presentation), and sexual orientation and race, ethnicity and racism.

Reporting on sex and gender	Human stool samples were collected for a previously published study (McPherson et al., Science Advances 2023; DOI: 10.1126/sciadv.add8766). Banked stool sample from the aforementioned study was used in our study. Sex and gender was not accounted for our study. Patient samples were selected based on diagnosis regardless of sex and gender.
Reporting on race, ethnicity, or other socially relevant groupings	Human stool samples were collected for a previously published study (McPherson et al., Science Advances 2023; DOI: 10.1126/sciadv.add8766). Banked stool sample from the aforementioned study was used in our study. Race, ethnicity, or other socially relevant groupings were not accounted for our study. Patient samples were selected based on diagnosis regardless of sex and gender.
Population characteristics	See above
Recruitment	Human stool samples were collected for a previously published study (McPherson et al., Science Advances 2023; DOI: 10.1126/sciadv.add8766). Banked stool sample from the aforementioned study was used in our study.
Ethics oversight	Human stool samples used in the flow cytometry test of human intelectin-2 were obtained under a protocol approved by the Massachusetts Institute of Technology [Institutional Review Board (IRB) protocol ID no. 1510271631]. The participants provided informed consent, and all experiments adhered to the regulations of the review boards.

Note that full information on the approval of the study protocol must also be provided in the manuscript.

Field-specific reporting

Please select the one below that is the best fit for your research. If you are not sure, read the appropriate sections before making your selection.

☒ Life sciences ☐ Behavioural & social sciences ☐ Ecological, evolutionary & environmental sciences

For a reference copy of the document with all sections, see nature.com/documents/nr-reporting-summary-flat.pdf

Life sciences study design

All studies must disclose on these points even when the disclosure is negative.

Sample size	Sample sizes were determined based on previous relevant studies.
Data exclusions	No data was excluded.
Replication	All experiments were conducted with at least two independent biological replicates. The number of replicates for each experiment is indicated in the figure legends. Once experiments and procedures were fully optimized, all replicates were successful.
Randomization	Sample allocations were random.
Blinding	NA

Reporting for specific materials, systems and methods

We require information from authors about some types of materials, experimental systems and methods used in many studies. Here, indicate whether each material, system or method listed is relevant to your study. If you are not sure if a list item applies to your research, read the appropriate section before selecting a response.

Materials & experimental systems

n/a	Involved in the study	
☐	☒	Antibodies
☐	☒	Eukaryotic cell lines
☒	☐	Palaeontology and archaeology
☐	☒	Animals and other organisms
☒	☐	Clinical data
☒	☐	Dual use research of concern
☒	☐	Plants

Methods

n/a	Involved in the study	
☒	☐	ChIP-seq
☐	☒	Flow cytometry
☒	☐	MRI-based neuroimaging

Antibodies

Antibodies used: hItln2 specific antibody (Bevins Lab, 1:5000); Goat-anti-rabbit HRP (Jackson, Cat# AB_2313567, 1:10,000); anti-StrepMAB-Classic HRP conjugate antibody (IBA, Cat# 2-1509-001, 1:10000); anti-StrepMAB-Classic DY549 antibody (IBA, Cat# 2-1566-050, 1:250)

Validation: Commercially available antibodies were validated by the manufacturers; anti-hItln2 antibody was validated by Bevins group.

Eukaryotic cell lines

Policy information about cell lines and Sex and Gender in Research

Cell line source(s): Cell lines used:
HEK 293T cells (University of Wisconsin-Madison)
Expi293F™ Cells (ThermoFisher)

Authentication: The cell lines were authenticated by the vendor. No additional authentication was performed.

Mycoplasma contamination: The cell lines were tested for mycoplasma upon receiving from manufacturer. No additional mycoplasma test were performed for these cells.

Commonly misidentified lines (See ICLAC register): No commonly misidentified cell lines were used in this study.

Animals and other research organisms

Policy information about studies involving animals; ARRIVE guidelines recommended for reporting animal research, and Sex and Gender in Research

Laboratory animals: Male BALB/cAnNTac mice, encoding a full Itln1-6 locus, were crossed with female C57BL/6NTac mice that encode a single intelectin gene, Itln1 (parent strains obtained from Taconic Biosciences, Germantown, NY). Male offspring, positive for the Itln1-6 locus (F1/N1: heterozygous), were crossed with pure C57BL/6NTac female mice for an additional nine generations (N10: ~99.9% C57BL/6NTac background). Small intestinal organoids were derived from mice approximately 10 weeks of age. All mice used in this work were housed in a temperature-controlled room with a 12 hrs light-dark cycle and were allowed free access to food and water in agreement with NIH animal care guidelines.

Wild animals: NA

Reporting on sex: Small intestinal tissue and derived small intestinal organoids were obtained from male animals.

Field-collected samples: NA

Ethics oversight: All animal experiments were approved by the Institutional Animal Care and Use Committee at the University of California, Davis.

Note that full information on the approval of the study protocol must also be provided in the manuscript.

Plants

Seed stocks	NA
Novel plant genotypes	NA
Authentication	NA

Flow Cytometry

Plots

Confirm that:

- [x] The axis labels state the marker and fluorochrome used (e.g. CD4-FITC).
- [x] The axis scales are clearly visible. Include numbers along axes only for bottom left plot of group (a 'group' is an analysis of identical markers).
- [x] All plots are contour plots with outliers or pseudocolor plots.
- [x] A numerical value for number of cells or percentage (with statistics) is provided.

Methodology

Sample preparation	Details of sample preparation are described in the methods section.
Instrument	ThermoFisher Attune NxT, BD Fortessa, and BD Symphony were used for flow cytometry.
Software	FlowJo (FlowJo™ v10.8 for Mac Software) was used for data analysis.
Cell population abundance	The abundance of lectin-binding cell population was quantified as a percentage of the total single cells, as described in the Methods section.
Gating strategy	Single cells were selected by using forward scatter (FSC-A/ FSC-H) and side scatter (SSC-A/ SSC-H) parameters. Furthermore, the gating for the population of interest was determined by comparison with different control samples, as described in the Methods section. Sample gating strategy is shown on Figure S4A.

- [x] Tick this box to confirm that a figure exemplifying the gating strategy is provided in the Supplementary Information.
